# *Burkholderia ambifaria* and *B. caribensis* Promote Growth and Increase Yield in Grain Amaranth (*Amaranthus cruentus* and *A. hypochondriacus*) by Improving Plant Nitrogen Uptake

**DOI:** 10.1371/journal.pone.0088094

**Published:** 2014-02-12

**Authors:** Fannie I. Parra-Cota, Juan J. Peña-Cabriales, Sergio de los Santos-Villalobos, Norma A. Martínez-Gallardo, John P. Délano-Frier

**Affiliations:** 1 Centro de Investigación y de Estudios Avanzados-Unidad Irapuato, Irapuato, Guanajuato, México; 2 Departamento de Ciencias del Agua y del Medio Ambiente, Instituto Tecnológico de Sonora, Ciudad Obregón, Sonora, México; University of Illinois at Chicago College of Medicine, United States of America

## Abstract

Grain amaranth is an emerging crop that produces seeds having high quality protein with balanced amino-acid content. However, production is restricted by agronomic limitations that result in yields that are lower than those normally produced by cereals. In this work, the use of five different rhizobacteria were explored as a strategy to promote growth and yields in *Amaranthus hypochondriacus* cv. Nutrisol and *A. cruentus* cv. Candil, two commercially important grain amaranth cultivars. The plants were grown in a rich substrate, high in organic matter, nitrogen (N), and phosphorus (P) and under greenhouse conditions. *Burkholderia ambifaria* Mex-5 and *B. caribensis* XV proved to be the most efficient strains and significantly promoted growth in both grain amaranth species tested. Increased grain yield and harvest index occurred in combination with chemical fertilization when tested in *A. cruentus*. Growth-promotion and improved yields correlated with increased N content in all tissues examined. Positive effects on growth also occurred in *A. cruentus* plants grown in a poor soil, even after N and P fertilization. No correlation between non-structural carbohydrate levels in roots of inoculated plants and growth promotion was observed. Conversely, gene expression assays performed at 3-, 5- and 7-weeks after seed inoculation in plants inoculated with *B. caribensis* XV identified a tissue-specific induction of several genes involved in photosynthesis, sugar- and N- metabolism and transport. It is concluded that strains of *Burkholderia* effectively promote growth and increase seed yields in grain amaranth. Growth promotion was particularly noticeable in plants grown in an infertile soil but also occurred in a well fertilized rich substrate. The positive effects observed may be attributed to a bio-fertilization effect that led to increased N levels in roots and shoots. The latter effect correlated with the differential induction of several genes involved in carbon and N metabolism and transport.

## Introduction

The genus *Amaranthus* L. (Caryophyllales: Amaranthaceae) comprises C4 dicotyledonous herbaceous plants classified into approximately 70 species having a relatively high level of genetic variability. They have a worldwide distribution, although most species predominate in the warm temperate and tropical regions of the world [1]. Many amaranth species are cultivated as ornamentals or as a source of highly nutritious pseudocereals (e.g. *grain amaranths*) and/or of vitamin- and mineral-rich leaf vegetables [2]–[4]. Others are notoriously aggressive weeds of commercial crops [5], [6]. The grain amaranths (predominantly *Amaranthus hypochondriacus* L., *A. cruentus* L., and *A. caudatus* L.) offer attractive nutritional and health-related traits (recently reviewed in [7] and [8]), in addition to many desirable agronomic characteristics. Thus, amaranth seeds are notable for their high contents of gluten-free protein possessing a nutritionally balanced amino-acid composition, the ability to release bioactive peptides when digested and relatively high levels of squalene-rich oil. Moreover, grain amaranths offer a viable alternative to cereals and other crops in agricultural settings where soil moisture conditions vary considerably between growing seasons [1]. Their ability to withstand drought and salt stress has been attributed to their superior water use efficiency [9]–[11], which is higher than other crops, including wheat, corn, cotton and sorghum [12]. Other contributing factors to abiotic stress resistance are the use the C4 pathway for CO_2_ fixation, an indeterminate flowering habit and the capacity to grow long taproots and develop an extensive lateral root system in response to water shortage in the soil [11], [13]. Osmolyte accumulation and the activation of stress-related genes are also associated with stress tolerance in grain amaranth [14], [15].

Augmented and consistent yields, increased pest resistance, and improved harvestability are important breeding goals that grain amaranth shares with all grain crops. The typical yields of current amaranth cultivars oscillate around ∼1000 kg/ha, although the potential exists for producing significantly higher yields that surpass 3000 kg/ha [1], [16]. If such potential could be more uniformly expressed, it should be possible to improve grain amaranth yields substantially [1].

Nowadays, several bio-fertilizers of bacterial or fungal origin are commercially available and may be utilized to improve productivity. In addition, they offer great ecological benefits associated with a number of properties that impinge positively on both the soil and the plants growing in it. These include the ability to fix atmospheric nitrogen, degrade organic compounds, including pesticides, and suppress various soil-borne pathogens via the synthesis of antibiotics, hydrogen cyanide and/or siderophores. Nitrogen fixation is of paramount importance considering that the natural supply of soil N usually limits plant yields in most agricultural cropping systems [17]. For this reason N fertilizer application is predicted to greatly increase in the next decades [18] unless N use efficiency (NUE) is significantly increased. NUE is defined as the total biomass or grain yield produced per unit of applied fertilizer N, and it integrates both Nitrogen uptake efficiency (the capacity of plant roots to acquire N from the soil) and Nitrogen utilization efficiency (the fraction of plant-acquired N to be converted to total plant biomass or grain yield) ([19] and references therein). Its importance is underlined by the deleterious effects that excess N compounds released from agricultural systems can have on the quality of air, water, and soil [17], [20].

Beneficial soil bacteria and fungi can also confer immunity against a wide range of foliar diseases and insects via the long-distance activation of plant defenses [21]. Growth promotion is believed to be tightly associated with the synthesis of bacterial auxins, giberellins and cytokinins, volatile compounds and/or vitamins, the induction of 1-aminocyclopropane-1-carbocylate (ACC) deaminase, which coupled to an increased superficial root area acting together with the secretion of siderophores, facilitate the absorption of limiting nutrients, such as iron, phosphorus and other minerals [22]–[28]. The bio-fertilizers of bacterial origin are commonly part of what are known as plant growth promoting rhizobacteria (PGPR). They constitute a large group on non-pathogenic soil bacteria that promote growth and/or control soil pathogens or insect pests when grown in a non-symbiotic association with plants [29]–[35]. Illustrative examples are the capacity to promote growth and protect against *Fusarium* wilts, in maize [36] and anthracnose, in mango [37] observed in plants inoculated with *Burkholderia cepacia*, and the induced systemic response against whitefly (*Bemisia tabaci*) pests detected in tomato plants inoculated with a *Bacillus subtilis* strain [38]. Other PGPR species belong to the *Rhizobium*, *Mesorhizobium* and *Bradyrhizobium* [39], [40], *Azospirillum* [41], *Agrobacterium*, *Azotobacter* [42], *Arthrobacter, Alcaligenes*, *Pseudomonas* [43], [44], *Serratia*, *Enterobacter, Beijerinckia, Klebsiella, Clostridium, Variovovax, Xanthomonas*, and *Phyllobacterium* genera [45]–[49]. Bio-fertilizers also include a group of phosphate-solubilizing microorganisms [50], [51] and certain mycoparasitic filamentous fungi of the genus *Trichoderma* [52]. Their use is considered to be innocuous both to man and the environment. They usually are more efficient in low-fertility soils and are economical and easily transported although care must be exercised to maintain their biological activity [47].

Information regarding the use of PGPR in *Amaranthus* is limited to reports that focused on growth promotion in leafy species [53], [54] and germination inhibition of weedy *A. hybridus* [55]. In addition, Nair and Anith [56] explored the use of PGPR for the control of leaf blight in *A. tricolor*. The comparative study herewith reported describes the effect that the inoculation of selected PGPR had on the growth and productivity of grain amaranth. Morphologic, metabolic and molecular studies were concomitantly performed in an effort to understand the possible mechanisms by means of which PGPR promote growth and increase yield and total biomass in grain amaranth. The information presented here has the potential to be employed to enhance the agronomic performance of grain amaranths in the field, while limiting N fertilizer application and thereby ameliorating the ecological damage associated to N pollution of the environment [57].

## Results

### Growth promotion experiments

The main objective of this work was to determine whether the utilization of PGPR with demonstrated agronomic potential was effective in promoting growth and increasing grain yield and total biomass in two species of grain amaranth. Biochemical and molecular tests were concomitantly performed to determine the possible mechanisms responsible for the changes observed.

The main characteristics of the five PGPR initially tested are shown in Table 1. They all showed at least one trait usually associated with plant growth promotion such as auxin production, ACC deaminase activity and siderophore production. The presence of acetylene reduction activity in the *Burkholderia* strains was indicative of the possible presence of nitrogenase activity required for nitrogen fixation.

**Table 1:** Properties of the rhizobacteria used in this study.

	*Rhizobium* sp. XXV^3^	*Bacillus subtilis* BEB-DN^2^	*Burkholderia ambifaria* Mex5^3^	*Burkholderia cepacia* XXVI^1^	*Burkholderia caribensis* XV^3^
Auxin production	+	+	+	−	−
ACC deaminase activity	−	+	+	−	+
Siderophore production	−	ND	+	+	+
Acetylene Reduction Activity	−	ND	+	+	+
Nitrogenase gene, *nifH 2*	ND	ND	ND	ND	ND

ND = Not determined.

1 = Described in Reference [37];

2 = Described in reference [91];

3 = Described in Reference [92].

**Figure 1:**
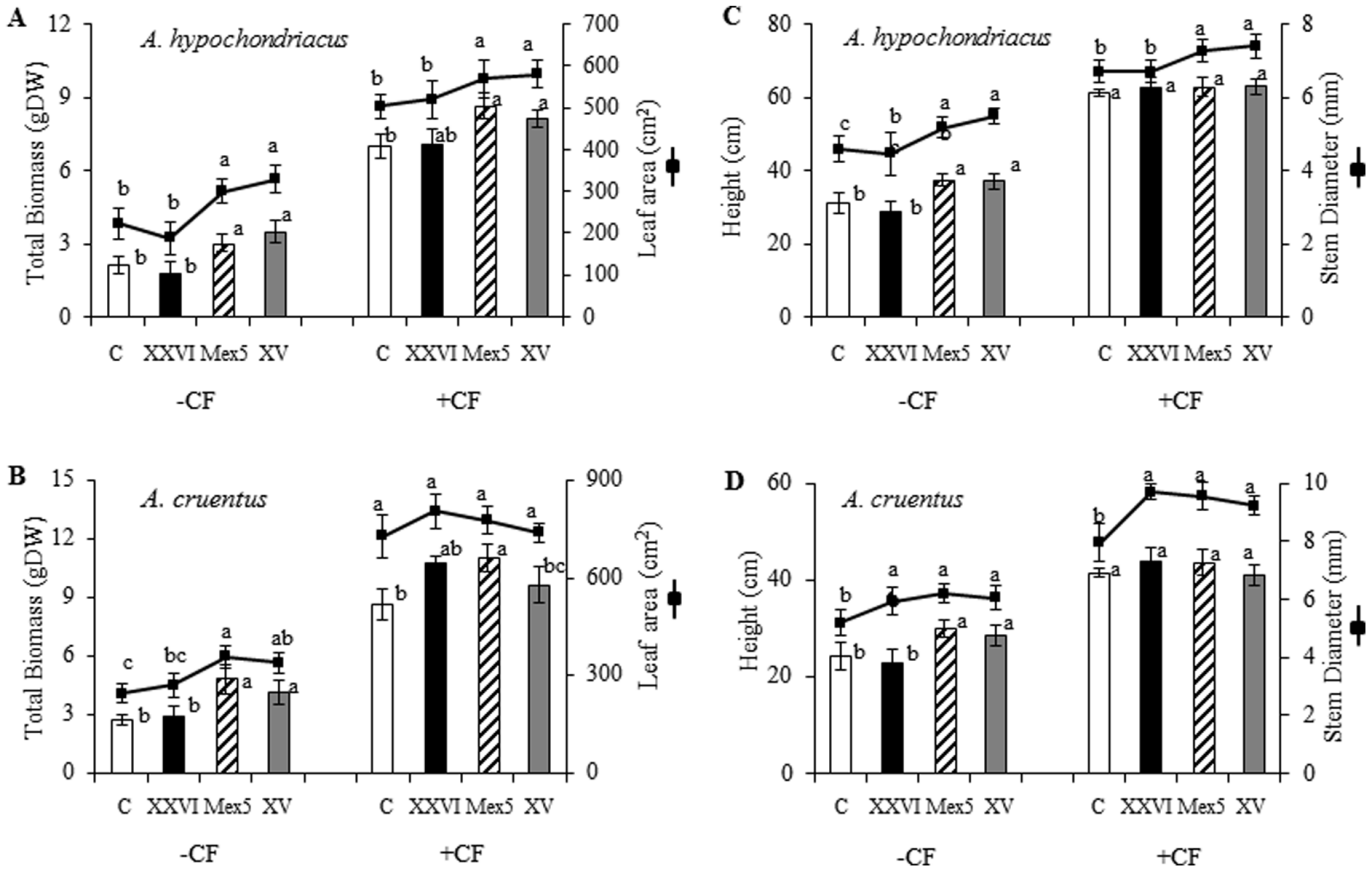
PGPR positively affect growth of grain amaranth plants. The effect of PGPR on different growth parameters, produced 8 weeks after inoculation with three strains of *Burkholderia* (*B. cepacia* XXVI, *B. ambifaria* Mex5 and *B. caribensis* XV), was determined in (**A**) and (**B**) *Amaranthus hypochondriacus* and (**C**) and (**D**) *A. cruentus* plants grown in a rich substrate, with (+CF) or without (−CF) chemical fertilization. Parameters measured were: total biomass, leaf area, plant height and stem diameter. Mean values ± SE are presented. Different letters over the bars and lines represent statistically different values at *P*≤0.05. Experiments were performed twice, and representative results are shown.

**Figure 2:**
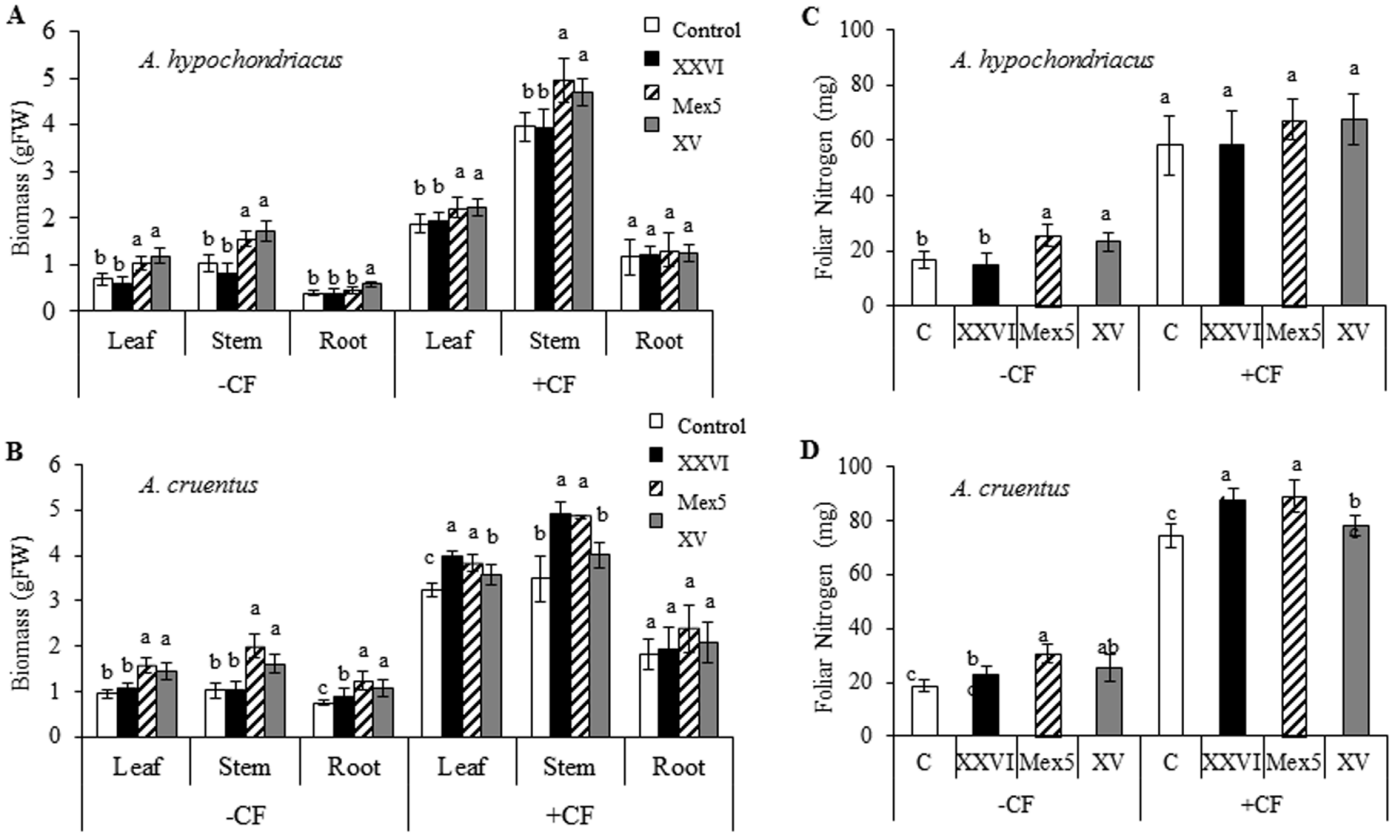
PGPR positively affect growth and nitrogen content of grain amaranth plants. The effect on the total biomass of leaves, stems and roots, (**A**) and (**B**), and on foliar nitrogen, (**C**) and (**D**), was measured 8 weeks after inoculation with three strains of *Burkholderia* (*B. cepacia* XXVI, *B. ambifaria* Mex5 and *B. caribensis* XV) in *Amaranthus hypochondriacus* and *A. cruentus* grown in a rich substrate, with (+CF) or without (−CF) chemical fertilization. Mean values ± SE are presented. Different letters over the bars and lines represent statistically different values at *P*≤0.05. Experiments were performed twice, and representative results are shown.

Initial exploratory experiments, performed in the commercially important *A. hypochondriacus* cv. Nutrisol and *A. cruentus* cv. Candil genotypes, showed that bacterial inoculation via direct seed-soaking produced better results than root drenching of seedlings. Growth promotion was determined 8 weeks post inoculation (wpi) by measuring total biomass, in general, and also separately in leaves, stems and roots. Plant height and stem diameter were two additional parameters determined. A positive effect was produced on both species and was observed with all PGPR tested, with the exception of *B. subtilus* BEB-DN (results not shown). Additional experiments were performed with those strains showing the best growth promoting efficiency, namely *B. caribensis* XV and *B. ambifaria* Mex-5, and to a lesser degree, *B. cepacia* XXVI. Significantly positive effects (Tukey test; P≤0.05, n = 5) on total biomass and leaf area, plant height and stem diameter (Figure 1), and leaf, stem and root biomass (Figure 2) were observed in both species at 8 wpi. The effect was still noticeable in plants that received chemical N and P fertilization, in particular in plants inoculated with *B. caribensis* XV and *B. ambifaria* Mex-5. It also tended to be more evident in *A. cruentus* than in *A. hypochondriacus* (Figures 1 and 2). Foliar nitrogen levels were also significantly higher in PGPR-treated *A. cruentus* and *A. hypochondriacus* plants (Figures 2C and D). For *A. cruentus*, this effect was evident even in plants subjected to N fertilization (Figure 2D).

**Figure 3:**
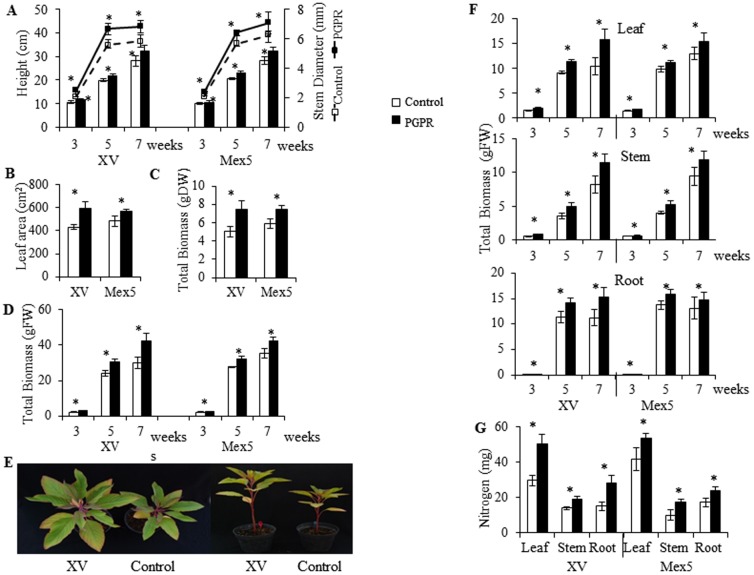
Time-course effect on different growth parameters produced in PGPR-inoculated amaranth plants. *A. cruentus* plants grown in a rich substrate were inoculated with two strains of *Burkholderia* (*B. ambifaria* Mex5 and *B. caribensis* XV) and growth-related parameters were measured at 3, 5 and 7 weeks after inoculation. These were (**A**) plant height and stem diameter; (**B**) leaf area; (**C**) and (**D**) total biomass in fresh and dry weight basis and (**F**) total leaf, stem and root biomass in a FW basis and (**G**) total foliar Nitrogen levels. Differences in plant height and leaf area produced between controls and plants inoculated with *B. caribensis XV* are shown in (**E**). Mean values ± SE are presented. Asterisks over the bars and lines represent statistically different values at *P*≤0.05. The results presented were obtained from a typical experiment that was repeated three times.

All further experiments were performed with *A. cruentus* cv. Candil which was inoculated with the best performing PGPR, i.e. *B. caribensis* XV and *B. ambifaria* Mex-5. The choice of *A. cruentus* was based on its relative insensitivity to the photo-period, a useful characteristic which allowed extended experimentation during early and late periods of the year, which are unsuitable for *A. hypochondriacus* [58]. All growth parameters tested were significantly increased in PGPR-inoculated *A. cruentus* plants (Tukey test; *P*≤0.05, n = 6), as shown for, stem diameter (average 23.6% [XV] and 18.5% [Mex5]) and plant height (average 17.6% [XV] and 16.9% [Mex5]) (Figure 3A). Increased leaf area (average 38.6%, [XV] and 23.1% [Mex5]) (Figure 3B) as well as total biomass in dry (average 55% and 29.3% increase for XV and Mex5, respectively) and fresh weight basis (Figures 3C and 3D), were also observed. The latter data were corroborated by significantly increased leaf (average 58.3% [XV] and 24.7% [Mex5]), stem (average 65.8% [XV] and 42.1% [Mex5]) and root (average 33.1% [XV] and 19.5% [Mex5]) biomass, which occurred during the entire duration of the experiment (Figure 3F). Total nitrogen content in the latter tissues was also significantly higher in PGPR-inoculated plants (Tukey test; *P*≤0.05, n = 6) (Figure 3G). The increments ranged from 27.7% (in leaves, [Mex5]) to 79.8% (in stems, [XV]). In general, growth promotion and NUE were more effective in plants inoculated with *B. caribensis* XV, as shown by the above data (see also Figure 3E).

After 7 weeks of growth, 1×10^6^ cfu per g substrate/soil were detected. This demonstrated the efficient long term colonization of the substrate/soil by *B. ambifaria* Mex5 and *B. caribensis* XV.

However, contrasting effects on the non-structural carbohydrates (NSC) levels in the different tissues examined were observed between the two PGPR tested. Inoculation with *B. caribensis* XV had a negative to neutral effect on all NSC tested and in all tissues examined, except for the late increase of hexoses in stem (GLC and FRC) and leaves (FRC) observed at 7 wpi (Table 2). On the other hand, and except for a few cases (i.e. starch in leaves at 3 wpi and FRC in stems, at 3 and 5 wpi), *A. cruentus* plants inoculated with *B. ambifaria* Mex-5 showed a biphasic oscillation in NSC levels, being neutral to negative at 3 and 5 wpi, and becoming predominantly positive at 7 wpi (Table 2). Thus, only growth promotion by *B. ambifaria* Mex-5 was associated with a gradual increase of NSC levels in roots and shoots.

**Table 2:** Time-course changes in non-structural carbohydrate levels in different tissues of PGPR-inoculated *A. cruentus* plants.

µmol/gFW								
			3 weeks		5 weeks		7 weeks	
Strain^1^	CHO	Tissue	Control	PGPR	Control	PGPR	Control	PGPR
**XV**	Starch	Leaf	20.97±0.85	19.37±1.42	43.19±2.93	41.51±1.63	51.76±2.55	46.22±1.09
		Stem	8.07±0.45	*5.44±0.65	33.85±3.27	31.51±2.80	52.09±3.28	47.28±2.91
		Root	ND	ND	1.25±0.14	1.24±0.09	2.51±0.21	2.56±0.20
	Sucrose	Leaf	1.67±0.09	1.51±0.08	3.91±0.47	3.54±0.24	2.93±0.11	3.00±0.21
		Stem	1.35±0.58	0.71±0.02	6.62±1.13	7.42±0.83	16.99±0.75	15.69±0.67
		Root	ND	ND	3.87±0.36	4.49±0.06	6.64±0.13	6.25±0.33
	Glucose	Leaf	7.49±0.51	*5.01±0.30	11.20±0.80	11.23±0.22	12.31±0.34	11.83±0.51
		Stem	5.57±1.04	4.51±0.05	30.62±4.01	26.77±1.88	22.50±0.69	*26.41±0.50
		Root	ND	ND	2.32±0.12	*0.99±0.14	1.94±0.19	*0.96±0.08
	Fructose	Leaf	2.00±0.13	*1.51±0.16	1.58±0.14	1.35±0.08	0.71±0.06	*1.13±0.15
		Stem	2.99±0.18	2.76±0.05	6.62±0.78	6.44±0.51	3.68±0.28	4.50±0.10
		Root	ND	ND	0.59±0.06	*0.45±0.02	0.44±0.04	*0.24±0.01
**Mex5**	Starch	Leaf	17.90±1.48	*23.53±1.45	41.49±2.45	*34.97±1.34	35.88±1.71	*48.99±3.50
		Stem	6.19±0.27	6.47±0.45	36.68±1.00	*32.32±1.46	42.83±3.72	*63.81±2.20
		Root	ND	ND	1.85±0.06	*1.03±0.05	2.60±0.17	*3.60±0.31
	Sucrose	Leaf	1.99±0.16	1.81±0.17	3.39±0.19	*2.24±0.09	2.13±0.06	*2.47±0.14
		Stem	0.61±0.04	*0.74±0.03	7.81±0.18	*6.82±0.24	15.86±0.63	14.98±0.99
		Root	ND	ND	3.67±0.04	*2.89±0.03	6.14±0.24	*7.76±0.39
	Glucose	Leaf	4.71±0.15	4.11±0.24	9.70±0.33	9.46±0.35	9.58±0.28	*11.35±0.39
		Stem	4.02±0.14	*4.91±0.14	31.32±0.42	*32.54±0.35	20.84±0.91	*28.41±0.88
		Root	ND	ND	2.52±0.24	*1.34±0.13	1.45±0.24	*2.97±0.23
	Fructose	Leaf	1.32±0.07	1.21±0.08	1.10±0.11	0.89±0.09	1.43±0.23	*0.71±0.09
		Stem	2.62±0.07	*3.19±0.05	7.54±0.08	*8.16±0.19	4.12±0.25	*5.50±0.50
		Root	ND	ND	0.63±0.04	*0.30±0.02	0.32±0.05	*0.61±0.07

1 = Burkholderia caribensis XV and B. ambifaria Mex5;

ND = Not determined;

* = Significant difference with controls at *P*<0.05.

**Figure 4:**
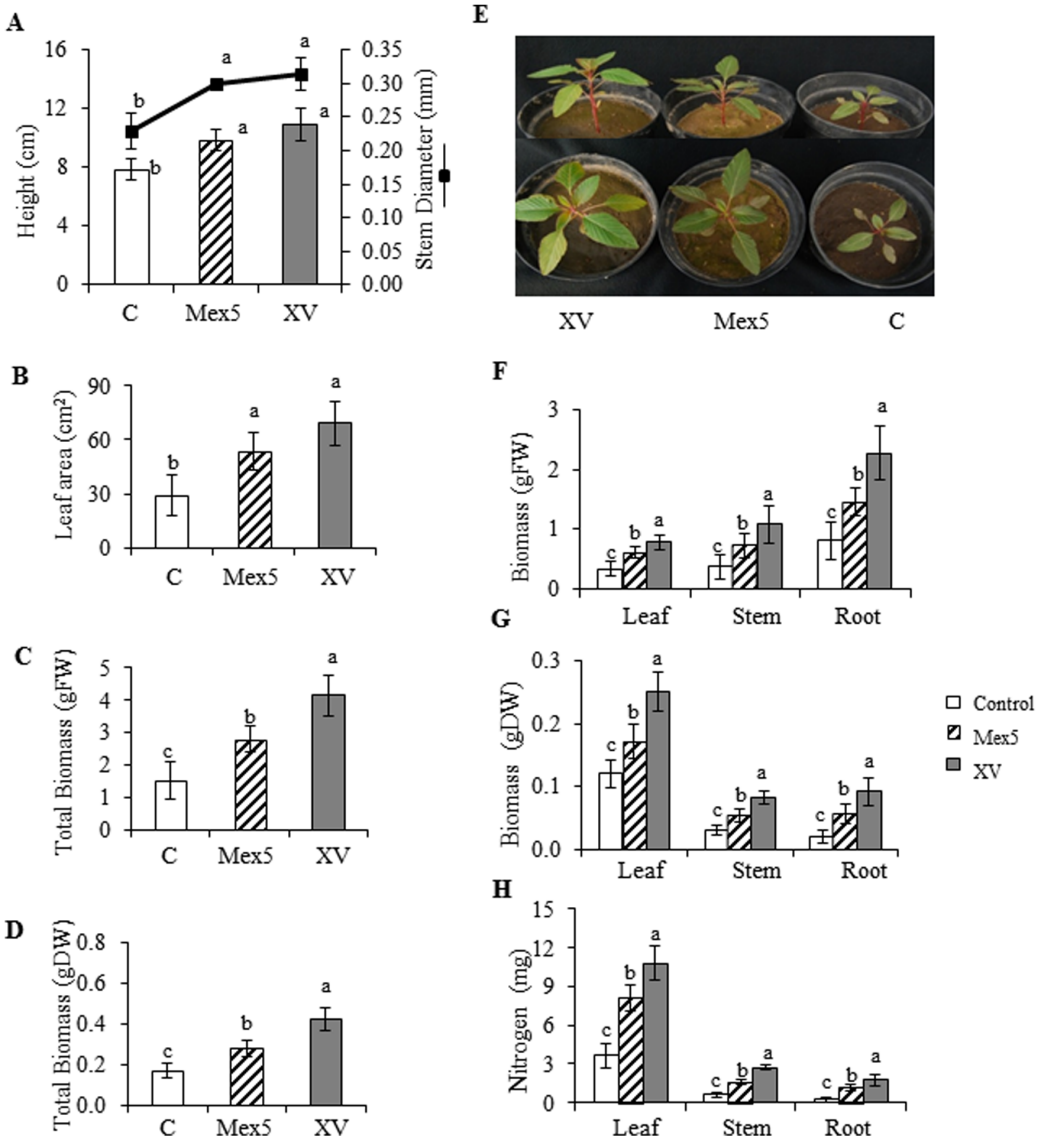
Growth promoting effect of PGPR inoculation on grain amaranth plants maintained in a low-fertility soil. The effect on different growth parameters were determined in *A. cruentus* plants grown in a low fertility soil and inoculated with two strains of *Burkholderia* (*B. ambifaria* Mex5 and *B. caribensis* XV). The parameters measured 7 weeks after inoculation were the following: **A**) plant height and stem diameter; **B**) leaf area; **C** and **D**) total biomass in fresh and dry weight basis, respectively, and **F** and **G**) total leaf, stem and root biomass in a FW and DW basis, respectively. Differences in plant height and leaf area between controls and plants inoculated with *B. caribensis XV* or *B. ambifaria* Mex5 are shown in (**E**). The effect on total nitrogen levels in leaves, stems and roots produced in plants inoculated with *B. caribensis* XV is shown in (**H**). Mean values ± SE are presented. Asterisks over the bars and lines represent statistically different values at *P*≤0.05. Experiments were performed twice, and representative results are shown.

**Figure 5:**
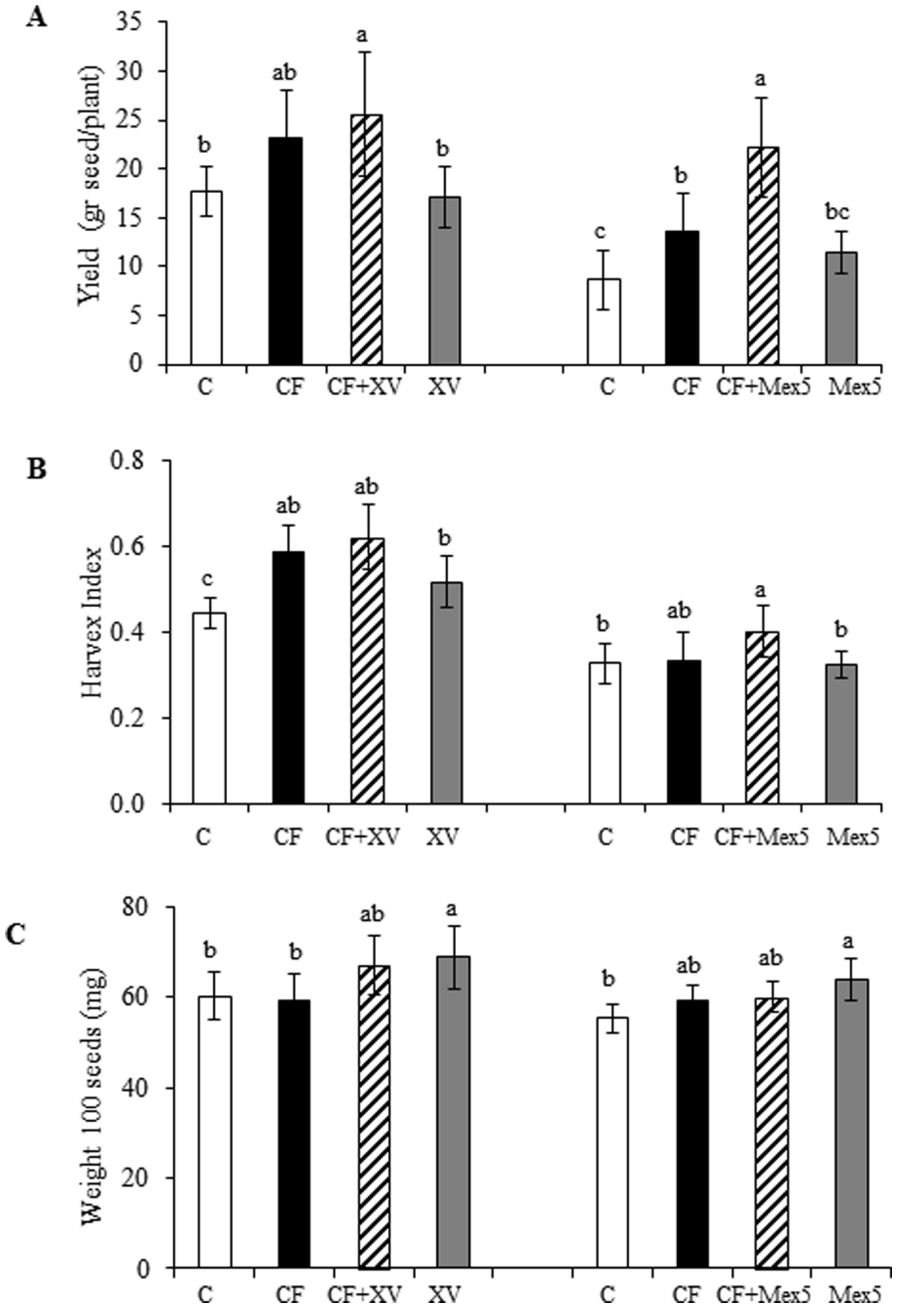
Effect on production parameters measured in *A. cruentus* plants inoculated with different PGPR. (**A**) Seed yield, (**B**) harvest index and (**C**) weight of 100 seeds were determined in *A. cruentus* plants inoculated with two strains of *Burkholderia* (*B. ambifaria* Mex5 or *B. caribensis* XV) and grown to maturity in a rich substrate. Inoculated plants ± chemical fertilization (CF) were compared with un-inoculated plants ± CF. Mean values ± SE are presented. Different letters over the bars represent statistically different values at *P*≤0.05. The results of a representative experiment that was performed in duplicate are shown.

The growth of *A. cruentus* plants was drastically reduced when they were grown in a poor soil, which contrasted with the rich substrate used in all other experiments by the much lower levels of fertility shown, predominantly in terms of organic matter and available N and P (Table S1). Inoculation with *B. caribensis* XV and *B. ambifaria* Mex5 significantly enhanced growth of *A. cruentus* plants as observed 7 wpi: stem diameter and plant height were increased between 29% (e.g stem diameter in Mex5) to 42% (e.g. plant height in XV) (Tukey test; *P*≤0.05, n = 7; Figure 4A).Leaf area and total biomass (in fresh and dry weight basis, respectively) were increased more than two-fold (Figures 4B to 5D). This was mirrored by the measurement of the leaf, stem and root biomasses, which were also significantly increased in inoculated plants. The effect was particularly evident in plants inoculated with *B. caribensis* XV (Figure 4E), in which the biomass of each tissue examined was more than doubled. In roots, for example, dry weigh biomass was augmented 5.6-fold (Tukey test; *P*≤0.05, n = 7; Figures 4F and G). Nitrogen content of inoculated plants grown in poor soil was also greatly increased. Increments ranged between 2- and 4- fold in leaves and roots of plants treated *B. caribensis* XV (Figure 4H). Similar results were obtained with *B. ambifaria* Mex5 (data not shown).

Taken together, the results demonstrated that the growth promotion induced by these two bacterial strains was increased on different substrates with different fertility levels, and was particularly striking in plants grown in a low fertility sandy soil, sampled in the Bajío region of central Mexico.

### Effects on yield, harvest index, and seed size

The results shown in Figure 5 indicate the effect that both *Burkholderia* strains employed had on the different production parameters tested. In contrast to the growth promotion experiments, *B. ambifaria* Mex5 was found to have a similar effect on production parameters as *B caribensis* XV. Yields were increased by chemical fertilization and the effect was significant in one repetition of the experiment (Tukey test; *P*≤0.05, n = 11; Figure 5A). However, in both cases, the combination with a bacterial partner significantly increased yields (41.4% for XV and 155.4% for Mex5, respectively). A similar tendency was observed when measuring the harvest index, although this parameter was increased solely by chemical fertilization and only marginal increases of 39.5% and 22.6% were detected when fertilized plants were treated together with *B. caribensis XV* and *B. ambifaria* Mex5, respectively (Tukey test; *P*≤0.05, n = 11; Figure 5B). Seed size was not affected by chemical fertilization; however, it was significantly increased (in a range of 7.2% to 15.5%) in the presence of the bacterial inoculates (Figure 5C).

### Gene expression assays

**Figure 6:**
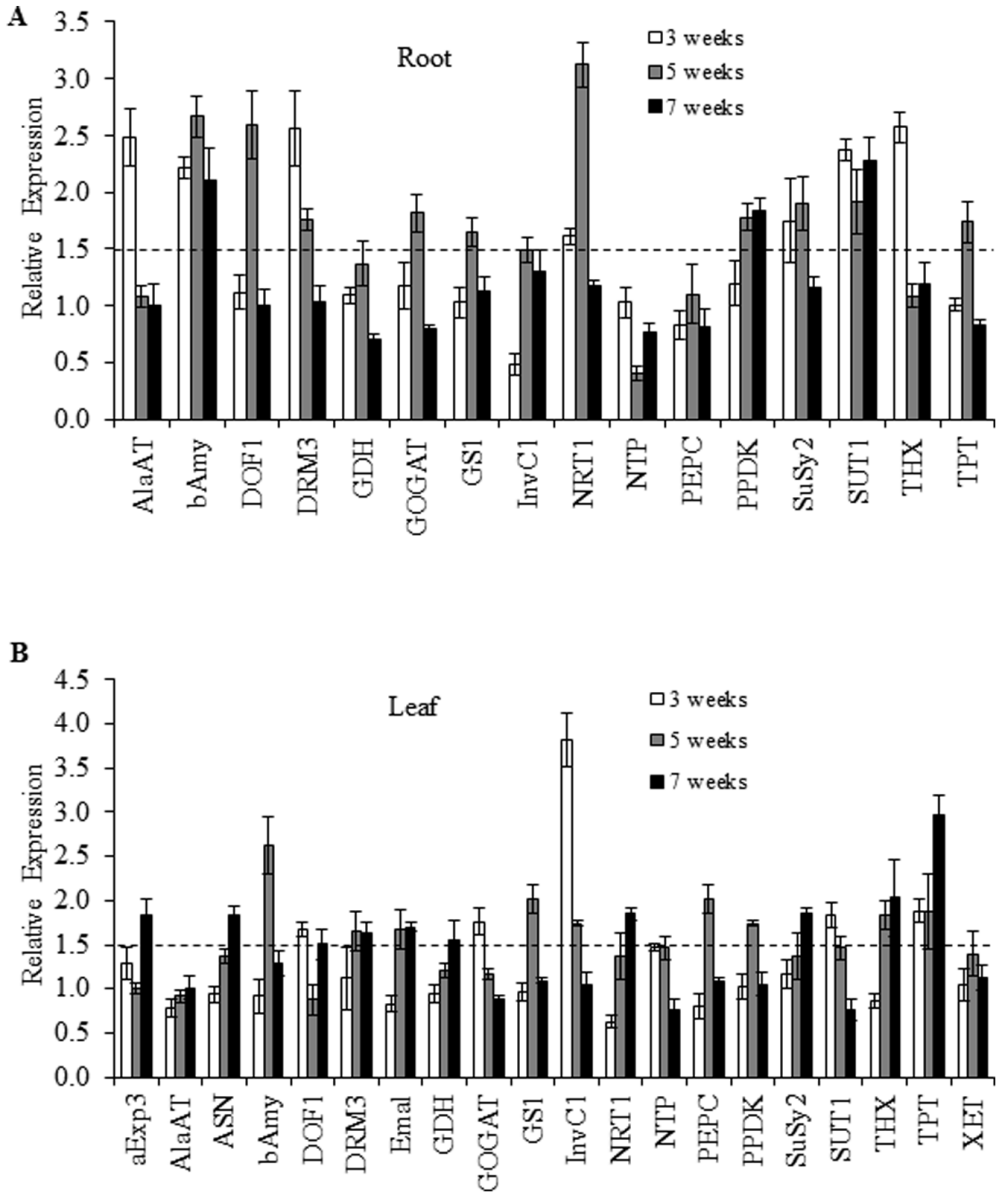
Real-time PCR analysis of gene expression in different tissues of PGPR-inoculated *A. cruentus* plants. The expression levels of a battery of genes involved in C and N metabolism and transport were measured in roots (**A**), and leaves (**B**), of *A. cruentus* plants inoculated with *Burkholderia caribensis* XV. The relative expression levels were determined by qPCR at 3, 5 and 7 weeks after seed inoculation, using the 2^−ΔΔCt^ method, as described in [94]. The bars represent mean values ± SE. Dashed lines indicate upper and lower limits beyond which genes were considered to be up- and down-regulated, respectively. Experiments were performed thrice, and results from a representative experiment are shown.

The results shown in Figure 6A showed that two genes remained up-regulated in roots of inoculated plants at the three sampling stages analyzed (3, 5 and 7 wpi). These coded for *AhBAMY* a ß-amylase and *AhSUT1*, a sucrose transporter. Others, such as *AhDRM3*, an auxin responsive gene, *AhSUS2*, sucrose synthase 2 and *AhNRT.1.1*, a nitrate transporter type 1.1 were up-regulated at 3 and 5 wpi but returned to basal levels at 7 wpi, whereas the pyruvate orthophosphate dikinase gene, *AhPPDK*, was induced at the latter stages of the process first appearing at 5 wpi and remaining up-regulated at 7 wpi. Other genes, including various associated with nitrogen metabolism were up-regulated at definite stages of the process. Such was the case of an alanine aminotransferase, *AhAlaAT* (up-regulated at 3 wpi), and NADH dependent glutamate synthase, *AhNADH GOGAT*, the *DOF1* transcription factor, *AhDOF1*, and cytosolic glutamine synthase 1, *AhGS1* (up-regulated at 5 wpi). A hexose transporter 1, *AhHT1* was also up-regulated early in the process (at 3 wpi), whereas a neutral cytosolic invertase1, *AhA/NI-1*, showed a contrasting pattern of expression, being repressed at 3 wpi but induced at 5 wpi.

The results obtained in leaves are shown in Figure 6B. The pattern differed amply from the gene expression patterns observed in roots. No gene was found to be expressed at all three stages examined. Genes that were up-regulated at only one time point were *AhDOF1*, *AhNADH GOGAT*, *AhSUT1* and a nitrate transporter 3 (*AhNRT.3*), that were induced at 3 wpi; *AhBAMY*, and two photosynthesis-related genes, namely phosphoenolenolpyruvate carboxylase, *AhPEPC*, and *AhPPDK*, were induced at 5 wpi, and α-expansin 3 (*AhEXP3α*), glutamine-dependent asparagine synthase 1 (*AhASN1*), *AhNRT.1.1* and *AhSUS2*, were induced at 7 wpi. All other genes were either expressed at 3 and 5 wpi (i.e. *AhA/NI-1*), 3 and 7 wpi (i.e. *Ah TPT*, a triose phosphate/phosphate transporter) and more frequently at 5 and 7 wpi (i.e. a NADPH-dependent malic enzyme malic enzyme, *AhME*, glutamate dehyrdrogenase 2, *AhGDH2*, *AhGS1*, *AhDRM3*, and *AhHT1*).

## Discussion

The growth promoting effects of rhizobacteria have the potential to be widely applied in agriculture, mainly as biofertilization, biocontrol and phytoremediation agents [59]–[61]. Several mechanisms are believed to be acting to permit these benefits, such as an enhanced nutrient uptake efficiency [19], hormone production or transformation [62], or improved defense against pathogens [60]. However, the molecular events underlying plant growth promotion by PGPR are still poorly understood. In this study, the long-term effect of diverse growth promoting rhizobacteria, including three potentially diazotrophic bacteria, on growth promotion, plant biomass accumulation and seed yield in grain amaranth were examined. In addition, changes in gene transcription and in sugar and nitrogen levels were also analyzed.

Grain amaranth is a marginal crop that has consistently attracted interest worldwide. This is mostly because grain amaranths can be utilized for the production of high quality grain in conditions that are unsuitable for cereal crops. However, there are many agronomic characteristics that must be improved (see [1]) in order to increase yields, which are much lower than those reported for cereal crops. Thus, it is imperative to develop appropriate agronomic practices for the cultivation of grain amaranth if higher yields are to be achieved.

Chemical fertilization is, together with optimal plant density, one key factor to boost grain amaranth yields, particularly when grown in poor or degraded soils. Up to a certain limit (≥90 Kg N/ha), amaranth grain yield is known to respond positively to nitrogen fertilizer, without increasing its tendency to lodge [63]–[65]. Also, nitrogen fertilization has been found to augment grain weight, biomass, grain yield and harvest index, although a negative effect was observed as nitrogen fertilizer rates were increased [66]. A similar effect was obtained in this study, since productivity was generally increased in fertilized *A. cruentus* plants. Interestingly, the beneficial effect observed was potentiated by inoculation with either *B. caribensis* XV or *B. ambifaria* Mex5 (see Figure 5).

The positive effect of N and P fertilization on grain amaranth's growth and biomass accumulation were also corroborated in this work, as shown by Figures 1 to 4, in which all parameters examined were significantly increased by N and P fertilization, including N uptake. Interestingly, and similarly to the productivity experiment, these parameters were also improved by the inoculation of the PGPR tested, even in chemically fertilized plants. Once again, this effect was particularly evident with two PGPR strains, namely *B. caribensis* XV and *B. ambifaria* Mex5. These are free-living and presumably diazotrophic *Burkholderia* strains that show promise for agro-biotechnological applications.

Growth promotion by inoculation with the *Burkholderia* strains was accompanied always by increased N levels in all plant tissues tested (i.e. roots, stems and leaves). It is therefore valid to propose that growth promotion effects were the result, at least partly, of an enhanced N uptake efficiency. This ‘biofertilization’ effect was consistent with the fact that, similarly to most crop plants, N availability is the main yield-limiting factor in grain amaranth (see above). The implied ability that these bacteria have to convert molecular nitrogen into ammonia by virtue of the nitrogenase enzyme complex ([67]; see Table 1), raised the possibility that the positive effects on plant growth and yield observed in grain amaranth were associated with an increased N provision occurring as a result of its fixation by the rhizobacterial partners. However, most experimental evidence gathered so far indicates that growth promotion by diazotrophic PGPR does not rely on the N_2_-fixation process, most probably because of its high energetic cost (see [59]). Other, more probable, scenarios have been raised in which stimulated plant growth is proposed to be the result of improved N nutrition occurring as a consequence of increased N uptake in the form of NO_3_^−^. This was in agreement with the results shown here, since growth promotion and grain yield were consistently shown to increase concomitantly with the N status of the substrate/soil in which grain amaranth was grown, being lowest in a low fertility soil deficient in N and NO_3_^−^ contents (see Table S1 and Figure 4) and highest in the rich substrate supplemented with chemical N and P (see Figures 1 to 3). The increased expression of the two nitrate transporters examined was in accordance with this possibility, considering the results of various expression studies that suggest that NO_3_^−^ uptake is primarily regulated at the transcriptional level (see below).

The experiments performed with *A. cruentus* cv. Candil (Figure 3) consistently showed that *B. caribensis* XV produced the best results in terms of growth promotion and grain yield. Curiously, plants inoculated with this strain showed no increase of sucrose (SUC) (in stem and roots) and starch (in leaves and stem) at 7 wpi (Table 2). These results have some similarity with a recent report in *Arabidopsis thaliana* showing that growth promotion resembled a sugar starvation-like transcriptional phenotype that was somehow induced by an unidentified signal from the associated bacterium [68]. These workers speculated that such response could be indicative of an increased metabolic demand for sugars and energy. Likewise, it could be proposed that the best gains in growth promotion and yield observed in *A. cruentus*, which were presumably caused by improved N uptake, occurred at the cost of a higher investment in C resources for the maintenance of the bacterial partner in the rhizosphere. More investigations are needed to prove this hypothesis.

However, the gene expression analysis was accordance with the above possibility. It showed that many genes involved in sugar transport and metabolism were up-regulated in response to the inoculation with *B. caribensis XV* in at least one sampling time point during the seven week period of experimentation. In roots, the sucrose transporter *AhSUT1* remained constantly up-regulated during this period, as well as *AhBAMY1*. Also relevant was the expression of a hexose transporter as well as a cytosolic invertase and an *AhSUS2* gene within the first five weeks after inoculation. The latter genes were also expressed in leaves, in addition to the *AhTPT* gene.

In the context of growth promotion, a previous report showed that the expression of *AtSUT1* and *AhBAM*Y1 were associated with the high tolerance to defoliation observed in grain amaranth. This report proposed that the up-regulation of these genes facilitated SUC transport and starch degradation in the early stages of plant recovery [69]. Additionally, the constitutive overexpression of a hexose transporter, STP13, in Arabidopsis, was shown to increase the expression of a high affinity nitrate transporter and total N uptake with the concomitant promotion of plant growth [70]. Moreover, the increased expression of cytosolic invertase1 and *AhSUS2* probably contributed to increase the hexose levels in order to fuel the observed growth promotion in roots and leaves. Importantly, the increased root surface area produced by the association with PGPR most probably enabled the plant to forage a larger volume of soil, which may have led to an enhanced nutrient uptake and consequent promotion of plant growth [59]. It could also be argued that increased transport and metabolism of sugars was probably supporting the augmented flow of C to the root-associated microorganisms present in the rhizosphere (reviewed in [71] and [72]).

In addition, the major role predicted for auxins in rhizobacterial growth promotion [62], [73] was supported by the expression in both leaves and roots of the *AhDRM3* gene. This gene was also found to be up-regulated in Arabidopsis plants inoculated with a naturally associated rhizobacterium [68]. The induction of the *AhDof1* gene in both roots and leaves of grain amaranth was in agreement with findings obtained from Arabidopsis and rice plants genetically engineered with a *Dof1* transcription factor, which showed better growth under N-limiting conditions and an enhanced net N assimilation, which was closely associated with the up-regulation of *PEPC, PPDK* (also induced in leaves, i.e. *AhPEPC*, and both leaves and roots, i.e. *AhPPDK*, of inoculated amaranth plants), and other genes coding for enzymes responsible for building the C skeletons used as platforms for inorganic N uptake [74], [75].

It is considered that despite their ability to fix atmospheric N_2_, diazotrophic PGPR are unlikely to provide large amounts of this form of N to the plants. However, they may greatly influence N nutrition by increasing NO_3_^−^ uptake capacity. One of the proposed mechanisms is by direct stimulation of NO_3_^−^ transport systems. The possibility that this mechanism was also responsible for the growth promotion observed in grain amaranth plants is supported by the expression of the two nitrate transporter genes examined in this study, most predominantly in roots. Such proposal is supported by numerous studies showing that NO_3_^−^ uptake is primarily regulated at the transcriptional level [76]–[79]. In addition, it was found that the constitutive expression of a high affinity nitrate transporter in rice led to the enhancement of vegetative growth under low nitrogen conditions [19].

The induction of genes involved in N assimilation was in accordance with the results obtained in a recent study in soybean whose aim was to identify genes associated with an enhanced nitrogen use efficiency [80]. Thus, similarly to this study, the gene expression analysis performed in inoculated grain amaranth showed that, in addition to genes involved in nitrate transport (see above), several other genes involved in N assimilation were induced in roots and/or leaves of grain amaranth inoculated with *B. caribensis XV*. These included genes coding for a glutamate dehydrogenase, an NADH GOGAT precursor, and an asparagine synthetase, which is known to be regulated by the carbon (C)/nitrogen (N) status of the plant. The expression of other genes involved in N assimilation, such as *AhGS1* and *AhAlaAT*, were also in accordance with several other related studies that have shown a positive correlation between the overexpression of cytosolic *GS1* and enhanced growth and/or yields in several plants species (reviewed in [19]) and with reports that demonstrated that the expression of a barley alanine aminotransferase gene in rice, led to significantly increased nitrogen uptake efficiency, biomass, and/or grain yields ([81], [82].

An increased expression of C4 photosynthesis-related genes, (*AhNADPH-ME, AhPEPC and AhPPDK*), mostly expressed in leaves, may have also indicated a need to increase CO_2_ uptake in order to sustain the enhanced plant growth produced by the association with the different PGPR tested. In this respect, various plant-microbe interactions have been previously described as having a strong effect on plant C metabolism [83], [84]. This may presumably represent an attempt by the bacteria to manipulate plant metabolism in order to gain access to nutrients, but may also be a manifestation of the positive growth effects of PGPR on plants.

## Conclusions

Grain amaranth is a highly tolerant species to adverse environmental conditions, including poor soils, lack of water and severe defoliation. However, grain amaranth production world-wide is hindered by relatively low yields. These are the consequence of several agronomic characteristics that negatively affect productivity [1]. This study demonstrated that both yield and biomass were significantly increased when grain amaranth plants were inoculated with free-living diazotrophic PGPR, which proved to be superior to other PGPR such as *B. subtilis* and *Rhizobium* spp. The effect was evident in both a rich substrate with high fertility and in an unfertile soil low in organic matter and primary nutrients, and was still relevant after chemical fertilization of the plants. Growth promotion appeared to be more evident in *A. cruentus* plants, particularly when inoculated with *B. caribensis XV*, a PGPR isolated from the rhizosphere of mango trees. An analysis of gene expression in *A. cruentus* plants inoculated with *B. caribensis XV* revealed that growth promotion was associated with the up-regulation of genes involved in C and N transport and metabolism. Thus, the application of PGPR to grain amaranth could be a strategy to improve productivity, particularly in poor soils with low fertility and could be also be employed to reduce chemical fertilization with the consequential reduction of the environmental pollution problems associated with excessive nitrogen fertilization.

## Methods

### Plant material and growth conditions

Seeds of *Amaranthus hypochondriacus* cultivar *Nutrisol* and *A. cruentus* cv. *Candil* were provided by Eduardo Espitia (INIFAP, México) and Universidad Nacional de La Pampa, Facultad de Agronomía (Argentina), respectively. The materials were chosen due to their commercial and agronomic importance in these countries. All experiments were performed in the greenhouse, under natural conditions of light and temperature, from mid-February to the end of November, which is the suitable growth season for grain amaranth in central Mexico.

### Bacterial growth conditions

The *Burkholderia* spp. strains were cultivated in LB medium [85]; *Bacillus subtilis* BEB-DN was cultivated in Potato Dextrose Broth as described previously [38], whereas *Rhizobium* sp. XVI was cultivated in LGI medium [86]. For inocula preparation, the bacteria were grown aerobically in 1.0-L to 1.5-L of the respective media (initial A_600_ = 0.1) on a rotary shaker (145 rpm) using 72 h incubation at 28°C to obtain bacteria in the exponential phase. The culture of bacterial cells was pelleted by centrifugation (5000×g, 7 min, 10°C), washed twice and re-suspended in sterile distilled-deionized water. To obtain 1×10^9^ colony forming units (cfu) per ml in the inoculum, the volume was adjusted based upon a correspondence established between the absorbance measured at 600 nm and the bacterial concentration. The density of bacteria was further estimated by plating dilutions of inoculum in Petri dishes containing 1.5% agar plus the respective medium (w/v). Bacteria were inoculated at a density of 1×10^6^ cfu/gr of substrate/soil.

### Bacterial re-isolation

Samples of 1 g of rhizospheric soil were collected 7 weeks after inoculation to determine the bacterial population of *B. ambifaria* Mex5 and *B. caribensis* XV. This was done following the methodology described by Constantino et al. [87]. The 16S rRNA gene sequences were determined by PCR amplification [88] and direct sequencing. For the phylogenetic analyses, related 16S rRNA gene sequences within the genus *Burkholderia* were included. 16S rDNA sequences were aligned by using the ClustalX program. The phylogenetic tree for the datasets was inferred from the neighbor-joining method described by Saitou and Nei [89] by using the Molecular Evolutionary Genetics Analysis (MEGA) software, version 5 [90] (data not shown).

### In planta screening for growth promotion

Briefly, in order to determine inoculation effects, two initial growth promotion pot experiments (GPPE) with two amaranth cultivars, five bacterial strains and two inoculation procedures were followed by three final GPPEs with one amaranth cultivar, two bacterial strains and one inoculation method. These experiments were performed in the years 2011 and 2012. In addition, a yield pot experiment (YPE) was performed with one amaranth cultivar, two bacterial strains and a mixed inoculation procedure in the summer/fall of 2012. In both the preliminary GPPEs and the YPE, the effect of chemical fertilization on PGPR bio-fertilization was evaluated. An additional comparative experiment was performed in the fall of 2012 with plants grown in a poor soil (GPPE-PS) collected from a field located in the town of San Juan de la Vega in the municipality of Celaya in the state of Guanajuato, Mexico (Table S1). No chemical fertilization was applied in this experiment.

The initial GPPE was performed (February to April, 2011) with both grain amaranth species and with five prospective growth promoting rhizobacterial strains having biocontrol properties. These were the following: *Bacillus subtilis* BEB-DN, originally isolated from the rhizosphere of field-cultivated potato plants in the municipality of León, state of Guanajuato, México [91] and known to confer resistance against whitefly infestation in tomato [38]; *Rhizobium spp.* XXV, *Burkholderia caribensis* XV, and *B. cepacia* XXVI, shown to be an effective biocontrol agent against anthracnose in mango fruits and isolated from the rhizosphere of mango trees growing in orchards located in the municipality of Apatzingán, State of Michoacán, México and Chauites, Oaxaca, México [37], [92]. *B. ambifaria* Mex-5 was isolated from teosinte plants (*Zea perennis*) growing in a natural reserve (“Reserva de la biósfera, Sierra de Manantlán”) located in the municipality of Autlán in the state of Jalisco, México. Other salient characteristics of these bacterial strains are shown in Table 1. Two inoculation procedures were tested: 1) seed soaking with bacterial cultures, for 30 min, when sowing in 2.5-L plastic pots and 2) soil application by drenching the base of the seedlings, three weeks after germination and at the moment of their transfer to 2.5-L pots. All inoculations were done with bacterial suspensions containing the equivalent of 1×10^6^ colony-forming units (cfu)/g of substrate. Inoculated seedlings had been previously germinated in 60-space germinating trays as described elsewhere [93]. The pots were filled with a sterile substrate composed of 3 parts Sunshine Mix 3™ (SunGro Horticulture, Bellevue, WA), 1 part loam, 2 parts mulch, 1 part vermiculite (SunGro Horticulture) and 1 part perlite (Termolita S.A., Nuevo León, México). The physicochemical characteristics of this rich substrate are shown in Table S1. All experiments were performed in greenhouses located at Cinvestav, Irapuato, México (20°40′18″N 101°20′48″W) under natural conditions of light and temperature. Several morphometric traits were measured in five plantlets per treatment at 8 weeks (soil drenching of 3 week-old seedlings) or 7 weeks (seed soaking at sowing) after inoculation. These were the following: plant height, stem diameter, total biomass (leaf, stem and roots, in both a dry [DWB] and fresh weight basis [FWB]) and leaf surface area. The latter was measured using a Portable Area Meter LI-3000 (Li-COR; Lincoln, NE, USA). The results of the first set of experiments, established the basis of a second one performed in September to November 2011, with both *A. cruentus* and *A. hypochondriacus*, in which only three bacterial strains (i.e. *B. caribensis* XV, *B. cepacia* XXVI and *B. ambifaria* Mex-5) were inoculated by seed soaking at sowing. In this second experiment, the performance of these PGPR was tested 8 weeks after germination in groups of five plants that included un-inoculated controls (± chemical fertilization) and inoculated controls (± chemical fertilization). Chemical fertilization was done by adding 1.25 g of N as (NH_4_)_2_SO_4_ and 0.857 g of P as P_2_O_5_ to the 2.5-L pots at the start of the experiments. The fertilization regime was based on the amount of N: P: K (180: 40: 00 Kg/ha) recommended for irrigated grain amaranth cultivation in Mexico (E Espitia-Rangel, personal communication).

### Pot experiments for growth promotion, nitrogen and carbohydrate content levels and variations in gene expression

Based on the above data, three additional GPPEs were performed in the late spring and summer of 2012 (May 7 to August 27). These experiments were performed under greenhouse conditions, as described above, and as follows: seeds of *A. cruentus* cv. Candil were soak-inoculated at sowing in 2.5-L plastic pots with 1×10^6^ CFU/g substrate of *B. caribensis* XV or *B. ambifaria* Mex-5. Plant height, stem diameter, leaf surface area, total biomass in both a FWB and DWB and leaf, stem and root biomass, in a FWB, were measured at 3, 5 and 7 weeks after sowing. Tissue sampling of six plants per treatment was performed at the same time points. The tissues sampled were leaf, stems and roots. They were stored at −80°C until required for the determination of total nitrogen content, non-structural carbohydrates (NSC) (starch, sucrose, glucose and fructose) levels and for gene expression analysis (see below).

### Pot experiments for seed yield, harvest index and weight of 100 seeds

Seeds of *A. cruentus* cv. Candil were soak-inoculated at sowing in 16-L plastic pots with 1×10^6^ CFU/g substrate of *B. caribensis* XV and *B. ambifaria* Mex-5. A second inoculation was performed 8 weeks after sowing by direct application to the substrate (1×10^6^ CFU/g) surrounding the roots. These experiments included groups of eleven plants comprising un-inoculated controls (± chemical fertilization), and inoculated plants (± chemical fertilization). This experiment was performed in the greenhouse under the above conditions, from May to November 2012. Step-wise harvest of the plants was started in late October and terminated in mid-November. Two replicates of the experiment were performed simultaneously. Colonization by *B. caribensis* XV and *B. ambifaria* Mex5 was corroborated in all experiments performed by collecting roots and isolating associated bacterial, as described above.

### Extraction of total RNA and cDNA preparation

Total RNA was extracted from 100–200 mg of frozen tissue with the Trizol reagent (Invitrogen, Carlsbad, CA, USA), according to the manufacturer's instructions, with modifications. These consisted of the addition of a salt solution (sodium citrate 0.8 M+1.2 M NaCl) during precipitation in a 1∶1 v/v ratio with isopropanol and further purification with LiCl (8 M) for one hour at 4°C. All RNA samples were analyzed by formaldehyde agarose gel electrophoresis and visual inspection of the ribosomal RNA bands upon ethidium bromide staining. Total RNA samples (1 µg for leaf and 3 µg for root) were reverse-transcribed to generate the first-strand cDNA using an oligo dT_20_ primer and 200 units of SuperScript II reverse transcriptase (Invitrogen).

### Gene expression analysis by quantitative real-time RT-PCR (qRT-PCR)

The cDNA employed for the qRT-PCR assays was initially prepared from 4 µg total RNA. It was then diluted ten-fold in sterile deionized-distilled (dd) water prior to qRT-PCR. Amplifications were performed using SYBR Green detection chemistry and run in triplicate in 96-well reaction plates with the CFX96 Real Time System (Bio-Rad, Hercules, CA, USA). Reactions were prepared in a total volume of 20 µl containing: 2 µl of template, 2 µl of each amplification primer (2 µM), 8 µl of IQ SYBR SuperMix (Bio-Rad) and 6 µl of sterile dd water. Quantitative real-time PCR was performed in triplicate for each sample using the primers listed in Table S2. Primers were designed for each gene, based on partial cDNA sequences derived from the transcriptomic analysis of *A. hypochondriacus* [93] or from complete cDNAs generated in a related study [69]. Primer design was performed using DNA calculator software (Sigma-Aldrich St. Louis, MO, USA) and included, when possible, part of unique 3′ non-coding regions to ensure specificity.

The following protocol was followed for all qRT-PCR runs: 15 min at 95°C to activate the *Taq* Polymerase, followed by 40 cycles of denaturation at 95°C for 15 s and annealing at 60°C for 1 min. Slow amplifications requiring an excess of 32 cycles were not considered for analysis. The specificity of the amplicons was verified by melting curve analysis after 40 cycles and agarose gel electrophoresis. Baseline and threshold cycles (Ct) were automatically determined using Real-Time PCR System software. PCR efficiencies for all genes tested were greater than 95%. Relative expression was calculated using the comparative cycle threshold method [94], where delta (Δ) cycle threshold of cDNA from un-inoculated controls was defined as 100% transcript presence.

The selection of genes was partly based on a recent report describing that the natural association of *A. thaliana* seedlings with growth promoting *Pseudomonas. sp.* G62 rhizobacteria induced a rapid and stable starvation-like transcriptional response which included genes involved in cell wall modification, C- and N-metabolism and auxin signaling [68]. These were *AhXET*, (xyloglucan endo-transglycosylase-related, isotig 04370), *AhEXP3α* (α-expansin 3, isotig 07296), *AhASN1* (Glutamine-dependent asparagine synthetase 1, isotig 11850), *AhGDH2* (Glutamate Dehydrogenase 2, isotig 09281), and *AhNRT.3* (Nitrate transporter 3, isotig 03624) and *DRM3*, an auxin responive gene (isotig 02637). Genes were also selected from a group of carbohydrate metabolism and C4 photosynthesis-related genes used to monitor changes in leaf gene expression in response to source-sink perturbation caused by partial shading of 12-month-old sugar cane plants [95]. These included the following: *AhME* (NADP-dependent malic enzyme, isotig 05148), *AhPEPC* (phosphoenolenolpyruvate carboxylase, isotig 16713), *Ah TPT* (triose phosphate/phosphate transporter, isotig 12255), and *AhHT* (hexose transporter, isotig 11515). Finally, genes involved in C mobilization and whose expression was positively correlated with defoliation tolerance in grain amaranth [69], were analyzed too. These included the following: *AhBAmy*1 (*β-amilase1*, isotig 03918); *AhA/NI-1* (*cytosolic invertase 1*; accession No. JQ012920), *AhSUT1* (sucrose transporter1, isotig 00313), and *AhSus2* (Sucrose synthase2, accession No. JQ012919). Genes were also selected on the basis of results obtained from transgenic approaches designed to improve plant nitrogen use efficiency (NUE) (reviewed in [19]). These included the following: *AhNRT.1.1* (nitrate transporter1.1; isotig 05430); *AhAlaAT* (alanine aminotransferase; contig 19731); *AhGS1* (cytosolic glutamine synthetase 1; isotig 04849); *AhNADH GOGAT* (NADH dependent glutamate synthase; isotig 12310); *AhDof1* (Dof1 transcription factor; isotig 15733), and *AhPPDK* (pyruvate orthophosphate dikinase; isotig 00544).

Transcript abundance data were normalized against the average transcript abundance of two reference genes: *actin* (isotig 10321) and *β-tubulin* (isotig 05486). These were obtained from the above transcriptomic study. The fold change in expression of the target genes in each treatment was calculated using the following equation: 2^−ΔΔCt^, where ΔΔCt = (Ct target gene - average Ct reference genes)_treatment_−(Ct target gene - average Ct reference genes)_control_ [94]. Values reported are the mean of three repetitions ± SE of one representative experiment. The qRT-PCR expression analysis was validated in three independent experiments.

### Determination of non-structural carbohydrate and nitrogen levels

All tissues (leaves, stems, roots and panicles) were collected at the beginning of the dark period (∼6:30 p.m.) and flash frozen in liquid nitrogen. Frozen ground tissue (200 mg) was extracted with 500 µl 80% aqueous ethanol (v/v) and incubated at 4°C for 10 min with stirring. After refrigerated centrifugation at 10,000 rpm (4°C for 10 min), the cleared supernatants were transferred into new tubes and concentrated by centrifugation (Heto Maxi Dry Lyo, Heto-Holten, Denmark). The residue was re-dissolved in 500 µl of 100 mM Hepes buffer, pH 7.4, and 5 mM MgCl_2_, and used for the determination of soluble sugars. The pellet derived from the centrifugation step was used for the determination of starch. To this end, it was homogenized with 500 µl of 10 mM KOH and incubated at 99°C for 2 h. Sucrose (SUC), glucose (GLC), fructose (FRC) and starch contents were measured using enzyme-based methods as instructed (Boehringer Mannheim/R-Biopharm, Darmstadt, Germany), except that the final reaction volume was reduced to fit a micro-plate format (250 µl per reaction).

Leaf N was determined by the micro-Kjeldahl method [96].

### Statistical analysis

All statistical analyses of the physiological and biochemical data were done using JPM8 at the *α* = 0.05 level (SAS Institute Inc., Cary, NC). Data were analyzed using an ANOVA. A Tukey test was performed with each ANOVA. In all figures, mean values and vertical bars representing standard errors (SE) are shown. In Table 2, standard errors are also listed beside mean values.

## Supporting Information

Table S1 Characteristics of the rich substrate and of the sandy, infertile soil used in the growth promotion experiments.
(DOCX)

Table S2 Primers used for gene expression analysis by qRT PCR.
(DOCX)

## References

[1] Brenner DM, Baltensperger DD, Kulakow PA, Lehmann JW, Myers RL, et al. (2000) Genetic resources and breeding of *Amaranthus*. In Janick J, editor. Plant Breeding Reviews. vol. 19. New York: John Wiley & Sons, Inc. pp 227–285.

[2] Hill RM, Rawate PD (1982) Evaluation of food potential, some toxicological aspects, and preparation of a protein isolate from the aerial part of amaranth (pigweed). J Agric Food Chem 30: 465–469. 7096800 10.1021/jf00111a014

[3] Shukla S, Bhargava A, Chatterjee A, Srivastava J, Singh N, et al (2006) Mineral profile and variability in vegetable amaranth (*Amaranthus tricolor*). Plant Foods Hum Nutr 61: 23–28. 16736385 10.1007/s11130-006-0004-x

[4] Akubugwo IE, Obasi NA, Chinyere GC, Ugbogu AE (2007) Nutritional and chemical value of *Amaranthus hybridus* L. leaves from Afikpo, Nigeria. Afr J Biotechnol 6: 2833–2839.

[5] Weaver SE, McWilliams EL (1980) The biology of canadian weeds: 44. *Amaranthus retroflexus* L., *A. powellii* S. Wats. and *A. hybridus* L. Can J Plant Sci 60: 1215–1234.

[6] Steckel LE (2007) The dioecious *Amaranthus* spp.: here to stay. Weed Technol 21: 567–570.

[7] Huerta-Ocampo J, Barba de la Rosa A (2011) Amaranth: a pseudo-cereal with nutraceutical properties. Curr Nutr Food Sci 7: 1–9.

[8] Caselato-Sousa VM, Amaya-Farfán J (2012) State of knowledge on amaranth grain: a comprehensive review. J Food Sci 77: R93–R104. 22515252 10.1111/j.1750-3841.2012.02645.x

[9] Li J, Wang S, Liu X, Li X, Gou J (1989) An observation of the root system growth of grain amaranth and its drought resistance. Agric Res Arid Areas 3: 34–41.

[10] Johnson BL, Henderson TL (2002) Water use patterns of grain amaranth in the northern Great Plains. Agron J 94: 1437–1443.

[11] Omami EN, Hammes PS, Robbertse PJ (2006) Differences in salinity tolerance for growth and water-use efficiency in some amaranth (*Amaranthus* spp.) genotypes. *N*ew Zeal J Crop Hort Sci 34: 11–22.

[12] Weber LE (1990) Amaranth grain production guide. New Crops Department, Rodale Research Center, Rodale Press, Emmaus, PA.

[13] Kadereit G, Borsch T, Weising K, Freitag H (2003) Phylogeny of Amaranthaceae and Chenopodiaceae and the evolution of C-4 photosynthesis. Int J Plant Sci 164: 959–986.

[14] Huerta-Ocampo JA, Briones-Cerecero EP, Mendoza-Hernandez G, De Leon-Rodriguez A, Barba de la Rosa AP (2009) Proteomic analysis of amaranth (*Amaranthus hypochondriacus* L.) leaves under drought stress. Int J Plant Sci 170: 990–998.

[15] Huerta-Ocampo JA, Leon-Galvan MF, Ortega-Cruz LB, Barrera-Pacheco A, De Leon-Rodriguez A, et al (2011) Water stress induces up-regulation of DOF1 and MIF1 transcription factors and down-regulation of proteins involved in secondary metabolism in amaranth roots (*Amaranthus hypochondriacus* L.). Plant Biol (Stuttg) 13: 472–482. 21489098 10.1111/j.1438-8677.2010.00391.x

[16] Myers R (1996) Amaranth: New crop opportunity. In: Janick J, editor. Progress in new crops. ASHS Press, Alexandria, VA. pp. 207–220.

[17] Robertson GP, Vitousek PM (2009) Nitrogen in agriculture: balancing the cost of an essential resource. Annu Rev Environ Resour 34: 97–125.

[18] Good AG, Shrawat AK, Muench DG (2004) Can less yield more? Is reducing nutrient input into the environment compatible with maintaining crop production? Trends Plant Sci 9: 597–605. 15564127 10.1016/j.tplants.2004.10.008

[19] Xu GH, Fan XR, Miller AJ (2012) Plant nitrogen assimilation and use efficiency. Annu Rev Plant Biol 63: 153–182. 22224450 10.1146/annurev-arplant-042811-105532

[20] Guo JH, Liu XJ, Zhang Y, Shen JL, Han WX, et al (2010) Significant acidification in major chinese croplands. Science 327: 1008–1010. 20150447 10.1126/science.1182570

[21] van Loon LC, Bakker PAHM, Pieterse CMJ (1998) Systemic resistance induced by rhizosphere bacteria. Annu Rev Phytopathol 36: 453–483. 15012509 10.1146/annurev.phyto.36.1.453

[22] Al-Taweil HI, Osman MB, Hamid AA, Wan Yussof WM (2009) Development of microbial inoculants and the impact of soil application on rice seedlings growth. Am J Agric Biol Sci 4: 79–82.

[23] Gamalero E, Glick BR (2011) Mechanisms used by plant growth-promoting bacteria. In: Maheshwari DK, editor. Bacteria in Agrobiology: Plant Nutrient Management. Springer-Verlag, Berlin Heidelberg. pp. 17–46.

[24] Kloepper JW (2003) A review of mechanisms for plant growth promotion by PGPR. In: Reddy MS, Anandaraj M, Eapen SJ, Sarma YR, Kloepper JW, editors. Abstracts and short papers. 6th International PGPR workshop, 5–10 october 2003. Indian Institute of Spices Research, Calicut, India. pp. 81–92.

[25] Suneja P, Dudeja SS, Narula N (2007) Development of multiple co-inoculants of different biofertilizers and their interaction with plants. Arch Agron Soil Sci 53: 221–230.

[26] Rengel Z, Marschner P (2005) Nutrient availability and management in the rhizosphere: exploiting genotypic differences. New Phytol 168: 305–312. 16219070 10.1111/j.1469-8137.2005.01558.x

[27] Ryu CM, Farag MA, Hu CH, Reddy MS, Wei HX, et al (2003) Bacterial volatiles promote growth in *Arabidopsis* . Proc Natl Acad Sci USA 100: 4927–4932. 12684534 10.1073/pnas.0730845100 PMC153657

[28] Zhang H, Kim MS, Krishnamachari V, Payton P, Sun Y, et al (2007) Rhizobacterial volatile emissions regulate auxin homeostasis and cell expansion in *Arabidopsis* . Planta 226: 839–851. 17497164 10.1007/s00425-007-0530-2

[29] Bashan Y, Holguin G (1998) Proposal for the division of plant growth-promoting rhizobacteria into two classifications: Biocontrol-PGPB (Plant Growth-Promoting Bacteria) and PGPB. Soil Biol Biochem 30: 1225–1228.

[30] Belimov AA, Safronova VI, Sergeyeva TA, Egorova TN, Matveyeva VA, et al (2001) Characterization of plant growth promoting rhizobacteria isolated from polluted soils and containing 1-aminocyclopropane-1-carboxylate deaminase. Can J Microbiol 47: 642–652. 11547884 10.1139/w01-062

[31] Kiely PD, Haynes JM, Higgins CH, Franks A, Mark GL, et al (2006) Exploiting new systems-based strategies to elucidate plant-bacterial interactions in the rhizosphere. Microb Ecol 51: 257–266. 16596439 10.1007/s00248-006-9019-y

[32] Pineda A, Zheng SJ, van Loon JJ, Pieterse CM, Dicke M (2010) Helping plants to deal with insects: the role of beneficial soil-borne microbes. Trends Plant Sci 15: 507–514. 20542720 10.1016/j.tplants.2010.05.007

[33] Vessey JK (2003) Plant growth promoting rhizobacteria as biofertilizers. Plant Soil 255: 571–586.

[34] Whipps JM (2001) Microbial interactions and biocontrol in the rhizosphere. J Exp Bot 52: 487–511. 11326055 10.1093/jexbot/52.suppl_1.487

[35] Zehnder GW, Murphy JF, Sikora EJ, Kloepper JW (2001) Application of rhizobacteria for induced resistance. Eur J Plant Pathol 107: 39–50.

[36] Bevivino A, Sarrocco S, Dalmastri C, Tabacchioni S, Cantale C, et al (1998) Characterization of a free-living maize-rhizosphere population of *Burkholderia cepacia*: effect of seed treatment on disease suppression and growth promotion of maize. FEMS Microbiol Ecol 27: 225–237.

[37] de los Santos-Villalobos S, Barrera-Galicia GC, Miranda-Salcedo MA, Pena-Cabriales JJ (2012) *Burkholderia cepacia* XXVI siderophore with biocontrol capacity against *Colletotrichum gloeosporioides* . World J Microbiol Biotechnol 28: 2615–2623. 22806187 10.1007/s11274-012-1071-9

[38] Valenzuela-Soto JH, Estrada-Hernandez MG, Ibarra-Laclette E, Delano-Frier JP (2010) Inoculation of tomato plants (*Solanum lycopersicum*) with growth-promoting *Bacillus subtilis* retards whitefly *Bemisia tabaci* development. Planta 231: 397–410. 20041333 10.1007/s00425-009-1061-9

[39] Khurana AL, Namdeo SL, Dudeja SS (1997) On-farm experiments on rhizobial inoculants: problems and possible solutions. In: Rupela OP, Johansen C, Herridge DF, editors. Managing legume nitrogen fixation in cropping systems of Asia: 20–24 Aug 1996; ICRISAT Asia Center. pp. 217–226.

[40] Khurana AL, Dudeja SS (1997) Biological nitrogen fixation technology for pulses interaction in India. Technical bulletin, Indian Institute of Pulses Research, Kanpur. pp. 1–18.

[41] Caballero-Mellado J, Carcaño Montiel M, Mascarua-Esparza M (1992) Field inoculation of wheat (*Triticum aestivum*) with *Azospirillum brasilense* under temperate climate. Symbiosis 13: 243–253.

[42] Narula N, Yadav KS (1989) Nitrogen fixation research in India with *Azotobacter*. In: Dadarwal KR, Yadav KS, editors. Biological nitrogen fixation research status in India. The Society for Plant Physiology and Biochemistry, New Delhi. pp. 87–124.

[43] Derylo M, Skorupska A (1993) Enhancement of symbiotic nitrogen-fixation by vitamin-secreting fluorescent *Pseudomonas* . Plant Soil 154: 211–217.

[44] Dudeja SS, Duhan JS (2005) Biological nitrogen fixation research in pulses with special reference to mungbean and urdbean. Indian J Pulses Res 18: 107–118.

[45] Defreitas JR, Germida JJ (1990) Plant-growth promoting rhizobacteria for winter-wheat. Can J Microbiol 36: 265–272.

[46] de Silva A, Patterson K, Rothrock C, Moore J (2000) Growth promotion of highbush blueberry by fungal and bacterial inoculants. Hort Sci 35: 1228–1230.

[47] Kloepper JW, Lifshitz R, Zablotowicz RM (1989) Free-living bacterial inocula for enhancing crop productivity. Trends Biotechnol 7: 39–44.

[48] Lucy M, Reed E, Glick B (2004) Applications of free living plant growth-promoting rhizobacteria. Antonie van Leeuwenhoek 86: 1–25. 15103234 10.1023/B:ANTO.0000024903.10757.6e

[49] Lugtenberg BJJ, Chin-A-Woeng TFC, Bloemberg GV (2002) Microbe-plant interactions: principles and mechanisms. Antonie Van Leeuwenhoek 81: 373–383. 12448736 10.1023/a:1020596903142

[50] Tiwari V, Pathak A, Lehri L (1993) Rock phosphate super-phosphate in wheat in relation to inoculation with phosphate solubilizing organisms and organic waste. Indian J Agric Res 27: 137–145.

[51] Toro M, Azcon R, Barea JM (1998) The use of isotopic dilution techniques to evaluate the interactive effects of *Rhizobium* genotype, mycorrhizal fungi, phosphate-solubilizing rhizobacteria and rock phosphate on nitrogen and phosphorus acquisition by *Medicago sativa* . New Phytol 138: 265–273. 10.1046/j.1469-8137.1998.00108.x 33863097

[52] Harman G, Howell C, Viterbo A, Chet I, Lorito M (2004) *Trichoderma* species-oportunistic, biological nitrogen fixation research in pulses. Nat Rev Microbiol 2: 43–56. 15035008 10.1038/nrmicro797

[53] Adesemoye AO, Torbert HA, Kloepper JW (2008) Enhanced plant nutrient use efficiency with PGPR and AMF in an integrated nutrient management system. Can J Microbiol 54: 876–886. 18923557 10.1139/w08-081

[54] Nair CB, Anith KN, Sreekumar J (2007) Mitigation of growth retardation effect of plant defense activator, acibenzolar-S-methyl, in *Amaranthus* plants by plant growth-promoting rhizobacteria. World J Microbiol Biotech 23: 1183–1187.

[55] Martinez-Mendoza EK, Mena-Violante HG, Mendez-Inocencio C, Oyoque-Salcedo G, Cortez-Madrigal H, et al (2012) Effects of *Bacillus subtilis* extracts on weed seed germination of *Sorghum halepense* and *Amaranthus hybridus* . Afric J Microbiol Res 6: 1887–1892.

[56] Nair C, Anith K (2009) Efficacy of acibenzolar-S-methyl and rhizobacteria for the management of foliar blight disease of amaranth. J Trop Agric 47: 43–47.

[57] Kraiser T, Gras DE, Gutierrez AG, Gonzalez B, Gutierrez RA (2011) A holistic view of nitrogen acquisition in plants. J Exp Bot 62: 1455–1466. 21239377 10.1093/jxb/erq425 PMC3137434

[58] Espitia-Rangel E, Mapes-Sánchez C, Escobedo-López D, de la O-Olán M, Rivas-Valencia P, et al.. (2010) Conservación y uso de los recursos genéticos de amaranto en México. SINAREFI-INIFAP-UNAM, Centro de Investigación Regional Centro, Celaya, Guanajuato, México. 201 p.

[59] Mantelin S, Touraine B (2004) Plant growth-promoting bacteria and nitrate availability: impacts on root development and nitrate uptake. J Exp Bot 55: 27–34. 14623902 10.1093/jxb/erh010

[60] van de Mortel JE, de Vos RCH, Dekkers E, Pineda A, Guillod L, et al (2012) Metabolic and transcriptomic changes induced in *Arabidopsis* by the rhizobacterium *Pseudomonas fluorescens* SS101. Plant Physiol 160: 2173–2188. 23073694 10.1104/pp.112.207324 PMC3510139

[61] Sheng XF, Xia JJ, Jiang CY, He LY, Qian M (2008) Characterization of heavy metal-resistant endophytic bacteria from rape (*Brassica napus*) roots and their potential in promoting the growth and lead accumulation of rape. Environ Pollut 156: 1164–1170. 18490091 10.1016/j.envpol.2008.04.007

[62] Persello-Cartieaux F, Nussaume L, Robaglia C (2003) Tales from the underground: molecular plant-rhizobacteria interactions. Plant Cell Environ 26: 189–199.

[63] Elbehri A, Putnam DH, Schmitt M (1993) Nitrogen-fertilizer and cultivar effects on yield and nitrogen-use efficiency of grain amaranth. Agron J 85: 120–128.

[64] Myers RL (1998) Nitrogen fertilizer effect on grain amaranth. Agron J 90: 597–602.

[65] Olaniyi JO, Adelasoye KA, Jegede CO (2008) Influence of nitrogen fertilizer on the growth, yield and quality of grain amaranth varieties. World J Agric Sci 4: 506–513.

[66] Thanapornpoonpong S (2004) Effect of nitrogen fertilizer on nitrogen assimilation and seed quality of amaranth (*Amaranthus* spp.) and quinoa (*Chenopodium quinoa* Willd). PhD Thesis. Georg-August-University of Göttingen, Sweden.

[67] Postgate JR (1982) Biological Nitrogen-Fixation: Fundamentals. Phil Trans R Soc Lond 296: 375–385.

[68] Schwachtje J, Karojet S, Thormählen I, Bernholz C, Kunz S, et al (2011) A naturally associated rhizobacterium of *Arabidopsis thaliana* induces a starvation-like transcriptional response while promoting growth. PLoS ONE 6 (12) e29382. 22216267 10.1371/journal.pone.0029382 PMC3247267

[69] Castrillón-Arbeláez PA, Martínez-Gallardo N, Avilés-Arnaut H, Tiessen A, Délano-Frier JP (2012) Metabolic and enzymatic changes associated with carbon mobilization, utilization and replenishment triggered in grain amaranth (*Amaranthus cruentus*) in response to partial defoliation by mechanical injury or insect herbivory. BMC Plant Biol 12: 163. 22966837 10.1186/1471-2229-12-163 PMC3515461

[70] Schofield RA, Bi YM, Kant S, Rothstein SJ (2009) Over-expression of *STP13*, a hexose transporter, improves plant growth and nitrogen use in *Arabidopsis thaliana* seedlings. Plant Cell Environ 32: 271–285. 19054349 10.1111/j.1365-3040.2008.01919.x

[71] Jones DL, Nguyen C, Finlay RD (2009) Carbon flow in the rhizosphere: carbon trading at the soil-root interface. Plant Soil 321: 5–33.

[72] Dennis PG, Miller AJ, Hirsch PR (2010) Are root exudates more important than other sources of rhizodeposits in structuring rhizosphere bacterial communities? FEMS Microbiol Ecol 72: 313–327. 20370828 10.1111/j.1574-6941.2010.00860.x

[73] Persello-Cartieaux F, David P, Sarrobert C, Thibaud MC, Achouak W, et al (2001) Utilization of mutants to analyze the interaction between *Arabidopsis thaliana* and its naturally root-associated *Pseudomonas* . Planta 212: 190–198. 11216839 10.1007/s004250000384

[74] Yanagisawa S, Akiyama A, Kisaka H, Uchimiya H, Miwa T (2004) Metabolic engineering with *Dof1* transcription factor in plants: Improved nitrogen assimilation and growth under low-nitrogen conditions. Proc Natl Acad Sci USA 101: 7833–7838. 15136740 10.1073/pnas.0402267101 PMC419692

[75] Kurai T, Wakayama M, Abiko T, Yanagisawa S, Aoki N, et al (2011) Introduction of the *ZmDof1* gene into rice enhances carbon and nitrogen assimilation under low-nitrogen conditions. Plant Biotechnol J 9: 826–837. 21624033 10.1111/j.1467-7652.2011.00592.x

[76] Forde BG (2000) Nitrate transporters in plants: structure, function and regulation. Biochim Biophys Acta 1465: 219–235. 10748256 10.1016/s0005-2736(00)00140-1

[77] Vidmar JJ, Zhuo D, Siddiqi MY, Schjoerring JK, Touraine B, et al (2000) Regulation of high-affinity nitrate transporter genes and high-affinity nitrate influx by nitrogen pools in roots of barley. Plant Physiol 123: 307–318. 10806247 10.1104/pp.123.1.307 PMC59004

[78] Glass ADM, Britto DT, Kaiser BN, Kinghorn JR, Kronzucker HJ, et al (2002) The regulation of nitrate and ammonium transport systems in plants. J Exp Bot 53: 855–864. 11912228 10.1093/jexbot/53.370.855

[79] Nazoa P, Vidmar JJ, Tranbarger TJ, Mouline K, Damiani I, et al (2003) Regulation of the nitrate transporter gene *AtNRT2.1* in *Arabidopsis thaliana*: responses to nitrate, amino acids and developmental stage. Plant Mol Biol 52: 689–703. 12956537 10.1023/a:1024899808018

[80] Hao QN, Zhou XA, Sha AH, Wang C, Zhou R, et al (2011) Identification of genes associated with nitrogen-use efficiency by genome-wide transcriptional analysis of two soybean genotypes. BMC Genomics 12: 525. 22029603 10.1186/1471-2164-12-525 PMC3210170

[81] Good AG, Johnson SJ, De Pauw M, Carroll RT, Savidov N (2007) Engineering nitrogen use efficiency with alanine aminotransferase. Can J Bot 85: 252–262.

[82] Shrawat AK, Carroll RT, DePauw M, Taylor GJ, Good AG (2008) Genetic engineering of improved nitrogen use efficiency in rice by the tissue-specific expression of alanine aminotransferase. Plant Biotechnol J 6: 722–732. 18510577 10.1111/j.1467-7652.2008.00351.x

[83] Biemelt S, Sonnewald U (2006) Plant-microbe interactions to probe regulation of plant carbon metabolism. J Plant Physiol 163: 307–318. 16368160 10.1016/j.jplph.2005.10.011

[84] Chen LQ, Hou BH, Lalonde S, Takanaga H, Hartung ML, et al (2010) Sugar transporters for intercellular exchange and nutrition of pathogens. Nature 468: 527–532. 21107422 10.1038/nature09606 PMC3000469

[85] Weaver VB, Kolter R (2004) *Burkholderia* spp. alter *Pseudomonas aeruginosa* physiology through iron sequestration. J Bacteriol 186: 2376–2384. 15060040 10.1128/JB.186.8.2376-2384.2004 PMC412164

[86] Cavalcante VA, Dobereiner J (1988) A new acid-tolerant nitrogen-fixing bacterium associated with sugarcane. Plant Soil 108: 23–31.

[87] Constantino M, Gomez-Alvarez R, Alvarez-Solis JD, Geissen V, Huerta E, et al (2008) Effect of inoculation with rhizobacteria and arbuscular mycorrhizal fungi on growth and yield of *Capsicum chinense* Jacquin. J Agric Rural Dev Trop 109: 169–180.

[88] Lane DJ (1991) 16S/23S rRNA sequencing. In: Stackebrandt E, Goodfellow M, editors. Nucleic acid techniques in bacterial systematics. John Wiley and Sons, New York. pp. 115–175.

[89] Saitou N, Nei M (1987) The neighbor-joining method: a new method for reconstructing phylogenetic trees. Mol Biol Evol 4: 406–425. 3447015 10.1093/oxfordjournals.molbev.a040454

[90] Tamura K, Peterson D, Peterson N, Stecher G, Nei M, et al (2011) MEGA5: Molecular Evolutionary Genetics Analysis using maximum likelihood, evolutionary distance, and maximum parsimony methods. Mol Biol Evol 28: 2731–2739. 21546353 10.1093/molbev/msr121 PMC3203626

[91] Jiménez-Delgadillo M (1999) Evaluación y caracterización fisiológica de rizobacterias empleadas como posibles agentes de biocontrol. MSc Thesis. Cinvestav I.P.N., Unidad Irapuato; Irapuato, México.

[92] de los Santos-Villalobos S, Folter S, Délano-Frier J, Gómez-Lim M, Guzmán-Ortiz D, et al (2013) Growth promotion and flowering induction in mango (*Mangifera indica* L. cv “Ataulfo”) trees by *Burkholderia* and *Rhizobium* inoculation: morphometric, biochemical, and molecular events. *J* Plant Growth Regul 32: 615–627.

[93] Délano-Frier JP, Avilés-Arnaut H, Casarrubias-Castillo K, Casique-Arroyo G, Castrillón-Arbeláez PA, et al (2011) Transcriptomic analysis of grain amaranth (*Amaranthus hypochondriacus*) using 454 pyrosequencing: comparison with *A. tuberculatus*, expression profiling in stems and in response to biotic and abiotic stress. BMC Genomics 12: 363. 21752295 10.1186/1471-2164-12-363 PMC3146458

[94] Livak KJ, Schmittgen TD (2001) Analysis of relative gene expression data using real-time quantitative PCR and the 2^−ΔΔCt^ method. Methods 25: 402–408. 11846609 10.1006/meth.2001.1262

[95] McCormick AJ, Cramer MD, Watt DA (2008) Changes in photosynthetic rates and gene expression of leaves during a source-sink perturbation in sugarcane. Ann Bot 101: 89–102. 17942591 10.1093/aob/mcm258 PMC2701831

[96] Humphries E (1956) Mineral components and ash analysis. In: Peach K, Tracy M, editors. Modern methods of plant analysis. Springer Verlag, Berlin. pp. 468–502.

