# Supplementary material for: Burkholderia ambifaria and B. caribensis Promote Growth and Increase Yield in Grain Amaranth (Amaranthus cruentus and A. hypochondriacus) by Improving Plant Nitrogen Uptake
Source: PLoS One. 2014 Feb 12;9(2):e88094. doi: 10.1371/journal.pone.0088094 (PMC3922803; doi:10.1371/journal.pone.0088094)
Supplement: Table S1 — Characteristics of the rich substrate and of the sandy, infertile soil used in the growth promotion experiments. (DOCX) [file pone.0088094.s001.docx]

| **Table S1. Characteristics of the rich substrate and the sandy, infertile soil used in the growth promotion experiments.** | | | | |
| --- | --- | --- | --- | --- |
|  | **Rich Substrate** | **Status** | **Infertile soil** | **Status** |
| **Physical Characteristics** | | | | |
| pH | 6.61 | Neutral | 7.81 | Mod. alkaline |
| Field capacity | 285% | - | 19.5% | - |
| Saturation Point | 380% | Very high | 26% | Mod. high |
| Permanent wilting point | 150% | - | 10.2% | - |
| Apparent density | 0.30 g/ cm^3^ | - | 1.48 g/ cm^3^ | - |
| Sand | - | - | 76.12% | - |
| Silt | - | - | 13.12% | - |
| Clay | - | - | 10.76% | - |
| **Fertility** | | | | |
| Organic matter | **17.8%** | **Very high** | **0.13%** | **Very low** |
| Inorganic Nitrogen (N) | **54.8 ppm** | **High** | **19.7 ppm** | **Average** |
| Available Phosphorus (Bray) | **38.2 ppm** | **Mod. high** | **3.74 ppm** | **Very low** |
| K | 463 ppm | Mod. high | 232 ppm | Average |
| Ca | 7196 ppm | Very high | 2933 ppm | Mod. High |
| Mg | 89.3 ppm | Low | 169 ppm | Mod. low |
| Na | 192 ppm | Average | 171 ppm | Average |
| Fe | **96.6 ppm** | Very high | **2.54 ppm** | **Very low** |
| Zn | **15.6 ppm** | Very high | **0.17 ppm** | **Very low** |
| Mn | **153 ppm** | Very high | **5.29 ppm** | **Mod. low** |
| Cu | **8.58 ppm** | Very high | **0.15 ppm** | **Very low** |
| N-NO_3_^-^ | 24.7 ppm | - | 21.8 ppm | - |
| **Saturation Extract (Salinity-sodicity)** | | | | |
| Electrical conductivity | 1.04 dS/m | - | 0.52 dS/m | - |
| Sodium Adsorption Ratio | 3.18 | Mod. Low | 4.34 | Mod. Low |
| Interchangeable sodium percentage | 2.14 | Low | 4.25 | Average |
| pHe | 6.95 | - | 7.98 | - |
| Ca^2+^ | 4.58 meq/ L |  | 1.70 meq/ L | - |
| Mg^2+^ | 5.34 meq/ L |  | 1.58 meq/ L | - |
| Na^+^ | 0.78 meq/ L |  | 2.08 meq/ L | - |
| K^+^ | 0.23 meq/ L |  | 0.06 meq/ L | - |
| Cl^-^ | 2.42 meq/ L |  | 1.52 meq/ L | - |
| HCO_3_^-^ | 1.14 meq/ L |  | 1.86 meq/ L | - |
| CO_3_^2-^ | 0.01 meq/ L |  | 0.01 meq/ L | - |
| SO_4_^2-^ | 6.86 meq/ L |  | 1.85 meq/ L | - |
| N-NO_3_^-^ | 0 meq/ L |  | 0 meq/ L | - |
| P-PO_4_^3-^ | 0 meq/ L |  | 0 meq/ L | - |
| **Extractable bases** | | | | |
| Ca^2+^ | 36 meq/ 100 g |  | 14.7 meq/ 100 g | - |
| Mg^2+^ | 0.73 meq/ 100 g |  | 1.39 meq/ 100 g | - |
| Na^+^ | 0.83 meq/ 100 g |  | 0.74 meq/ 100 g | - |
| K^+^ | 1.18 meq/ 100 g |  | 0.59 meq/ 100 g | - |
| Cation exchange capacity | 38.7 meq/ 100 g |  | 7.4 meq/ 100 g | - |
