# Supplementary material for: Burkholderia ambifaria and B. caribensis Promote Growth and Increase Yield in Grain Amaranth (Amaranthus cruentus and A. hypochondriacus) by Improving Plant Nitrogen Uptake
Source: PLoS One. 2014 Feb 12;9(2):e88094. doi: 10.1371/journal.pone.0088094 (PMC3922803; doi:10.1371/journal.pone.0088094)
Supplement: Table S2 — Primers used for gene expression analysis by qRT PCR. (DOCX) [file pone.0088094.s002.docx]

**Table S2. Primers used for gene expression analysis by qRT PCR.**

| Gene |  | Sequence |
| --- | --- | --- |
| Glutamate dehydrogenase (*AhGDH*) | Forward | ATGCATTCCATACTTCCATTT |
|  | Reverse | ACACCGGAGTAGTTACACAATATTA |
|  |  |  |
| Asparagine synthetase (*AhAS*) | Forward | GCACATCTCGTTTCATTCAG |
|  | Reverse | GGGACACAAGTTACAACACAAG |
|  |  |  |
| Nitrate transporter (*AhNRT1.4*) | Forward | GAGGAGAAGTGCTAGCAGACTAC |
|  | Reverse | CAAGAATGACACCAAACATCA |
|  |  |  |
| Alanine aminotransferase (*AhAlaAT*) | Forward | ATTAAGGGACTGCGTGAAAC |
|  | Reverse | TGAAGCAGAATACAACGGAT |
|  |  |  |
| DOF transcription factor (*AhDOF1*) | Forward | GGCTATTTACACACCAGGTTC |
|  | Reverse | TAATACCACTAACCAAACGGAC |
|  |  |  |
| Glutamate synthase-NADH-dependent (*AhGOGAT*) | Forward | GCTGGCAGAGATATGAAGACTAT |
|  | Reverse | ATCTATCTTGGCCCCACTACC |
|  |  |  |
| Glutamine synthetase (*AhGS1*) | Forward | GCTGAGACAACTATCATCTGGA |
|  | Reverse | ATGCAGTACTAGATTCACCTTCC |
|  |  |  |
| Nitrate transporter (*AhNRT1.1*) | Forward | TGTGGAAGAAGAAGAAGATGG |
|  | Reverse | CTCTTAAATTATTGCACGGGTA |
|  |  |  |
| Pyruvate phosphate dikinase (*AhPPDK*) | Forward | GCTTGAAGGTTGGTATCTGTG |
|  | Reverse | CTAATTCATCTGGTGACTCGTG |
|  |  |  |
| Xyloglucan endotransglycosylase (*AhXET*) | Forward | ATCTTGATTCATACCAATATAGGAGA |
|  | Reverse | GTAGTTGGTAGTTGATGGTATTGATT |
|  |  |  |
| α Expansin **(***Ah****EXPA3***) | Forward | CAAGAGCTCAATAGTCATGC |
|  | Reverse | GTTAGTGTGACCGTGTAATTG |
|  |  |  |
| Phosphoenolpyruvate carboxylase (*AhPEPC*) | Forward | AACGCTGCTCGATATTTCTAAT |
|  | Reverse | TGACAATAATACAGCAGTAAGAAATC |
|  |  |  |
| NADPH Malic enzyme (*AhME*) | Forward | CAATGCAGGGTCTTCTCTAATAG |
|  | Reverse | ATTCAATGTGCATCATACGC |
|  |  |  |
| Triose-phosphate transporter (*AhTPT*) | Forward | AAGTCTGCTTGAGTTGATCGT |
|  | Reverse | TACAGATTGATAATTGTTCATTCTCC |
|  |  |  |
| β- Amylase (*AhBAM*) | Forward | GAGCTTACAAGTCACAATACCAC |
|  | Reverse | GCAAGCTCAAAAGGTTTATG |
|  |  |  |
| Cytosolic invertase (*AhCINV1*) | Forward | ATATTGCTTCTTCATTGATGTTTG |
|  | Reverse | TAATTTTATCgAACAATgTCTTTTgAA |
|  |  |  |
| Sucrose synthase (*AhSuSy2*) | Forward | GAAATCCGTACCTCTGGCATC |
|  | Reverse | CCTggCAAAAgTATTCCgAAA |
|  |  |  |
| Sucrose transporter (*AhSUT1*) | Forward | CTCgACgTAgCCAACAACACTCTTC |
|  | Reverse | GTGAATGGGAATATTTTGTAGAGACGG |
|  |  |  |
| Hexose transporter (*AhHXT*) | Forward | GTGTTATTCTTAATGCTGCTGC |
|  | Reverse | CCGATTGTGACGTTGAGTT |
|  |  |  |
| Dormancy/auxin associated protein  (*AhDRM3*) | Forward | CCTTGCTACTGTCTAATATGTCG |
|  | Reverse | CCTATTACATTCACGGATTACAT |
|  |  |  |
| Actin (*AhATC*) | Forward | CGTGACCTGACTGATTACCTTA |
|  | Reverse | GCTCGTAGTTCTTCTCAATGGC |
|  |  |  |
| β-tubulin (*AhTUB*) | Forward | TCTCAGCAGTATGTCTCCCTCA |
|  | Reverse | TCTACTTCTTTGGTGCTCATCTT |
